# NIR-responsive cetuximab-functionalized chitosan–polydopamine hydrogel Co- delivering Ca^2+^ and gefitinib for EGFR-targeted esophageal cancer therapy via Ca^2+^- mediated cytotoxicity and potential immunomodulatory effects

**DOI:** 10.1186/s13036-026-00726-w

**Published:** 2026-07-22

**Authors:** Jing Chen, Shirong Wu, Yu Zhang, Ya Zhao, Mengqi Xiang, Liqiong Hao

**Affiliations:** 1 Department of Medical Oncology, Sichuan Clinical Research Center for Cancer, Sichuan Cancer Hospital & Institute, Sichuan Cancer Center, Affiliated Cancer Hospital of University of Electronic Science and Technology of China, Chengdu, 610041 China; 2 Department of Thoracic Surgery, Sichuan Clinical Research Center for Cancer, Sichuan Cancer Hospital & Institute, Sichuan Cancer Center, Affiliated Cancer Hospital of University of Electronic Science and Technology of China, Chengdu, 610041 China; 3 Department of Nursing Care, Sichuan Clinical Research Center for Cancer,Sichuan Cancer Hospital & Institute, Sichuan Cancer Center, Affiliated Cancer Hospital of University of Electronic Science and Technology of China, Chengdu, China; 4 Department of Medical Oncology, Sichuan Cancer Hospital, Medical School of University of Electronic Science and Technology of China, No.55, Section 4, South Renmin Road, Chengdu, 610041 China

**Keywords:** Esophageal cancer, Gefitinib, Cetuximab, Cytotoxicity

## Abstract

The current study presents the development of a Cetuximab (CTX)-functionalized chitosan–polydopamine (CS–PDA) hydrogel co-delivering calcium ions (Ca^2 +^) and Gefitinib (GFT) for EGFR-targeted therapy against esophageal cancer through the induction of Ca^2+^ mediated cell death and potential immune modulatory effects. FTIR analysis confirmed the functional groups present in the fabricated hydrogel, while UV–Vis spectroscopy verified successful drug incorporation, and SEM analysis revealed a porous morphology suitable for efficient drug encapsulation. The hydrogel shows high drug loading efficiencies of 78.5 ± 2.3% for Ca^2 +^ and 81.6 ± 3.1% for GFT, with pH-responsive drug release reaching up to 70% under acidic conditions. NIR-activated CTX-CS-pDA@Ca^2 +^/GFT hydrogel showed superior cytotoxicity in KYSE-140 cells, with significantly lower IC₅₀ values than free GFT. Further, JC-1 and AO/EB staining confirmed mitochondrial depolarization and apoptosis induction, while flow cytometry demonstrated that CTX-CS-pDA@Ca^2 +^/GFT + NIR markedly induced apoptosis in KYSE-140 cells by combining EGFR inhibition, Ca^2 +^ -mediated stress, and NIR-enhanced ROS generation, resulting in reduced cell viability and increased apoptotic populations. Calcium imaging further validated intracellular Ca^2 +^ accumulation indicated enhanced Ca^2+^mediated cellular stress in treated cells. In vivo evaluation in a xenograft mouse model revealed tumor inhibition rates of 42%, 24%, 60%, 78%, and 92% for GFT, CS-pDA, CS-pDA@Ca^2 +^/GFT, CTX-CS-pDA@Ca^2 +^/GFT, and CTX-CS-pDA@Ca^2 +^/GFT + NIR, respectively. The NIR-activated CTX-CS-pDA@Ca^2 +^/GFT formulation achieved the most pronounced tumor regression with negligible impact on body weight, confirming its superior therapeutic efficacy and biosafety. Biochemical analyses of serum ALP, SGOT, urea, and creatinine levels confirmed the biocompatibility and safety of the fabricated hydrogel. Histopathological examination of excised tumor tissues supported the induction of apoptosis and tissue necrosis in treated groups. Furthermore, qRT-PCR and Western blot analyses demonstrated upregulation of Bax and cleaved caspase-3 and downregulation of Bcl-2 and EGFR/p-AKT signaling, supporting the dual mechanism of action. Besides, CTX-CS-pDA@Ca^2 +^/GFT + NIR significantly upregulated pro-inflammatory cytokines (IL-6, TNF-α, and IFN-γ), indicating potential immunomodulatory activity associated with cytokine expression changes driven by targeted delivery, Ca^2 +^ -mediated stress, and NIR-triggered activation. Overall, this multifunctional hydrogel demonstrates strong potential as a localized and targeted therapeutic strategy for EGFR-overexpressing esophageal cancer through synergistic EGFR inhibition, Ca^2 +^ -mediated cytotoxicity, and potential immunomodulatory effects.

**Supplementary Information:**

The online version contains supplementary material available at https://doi.org/10.1186/s13036-026-00726-w.

## Graphical Abstract

**Figure:**
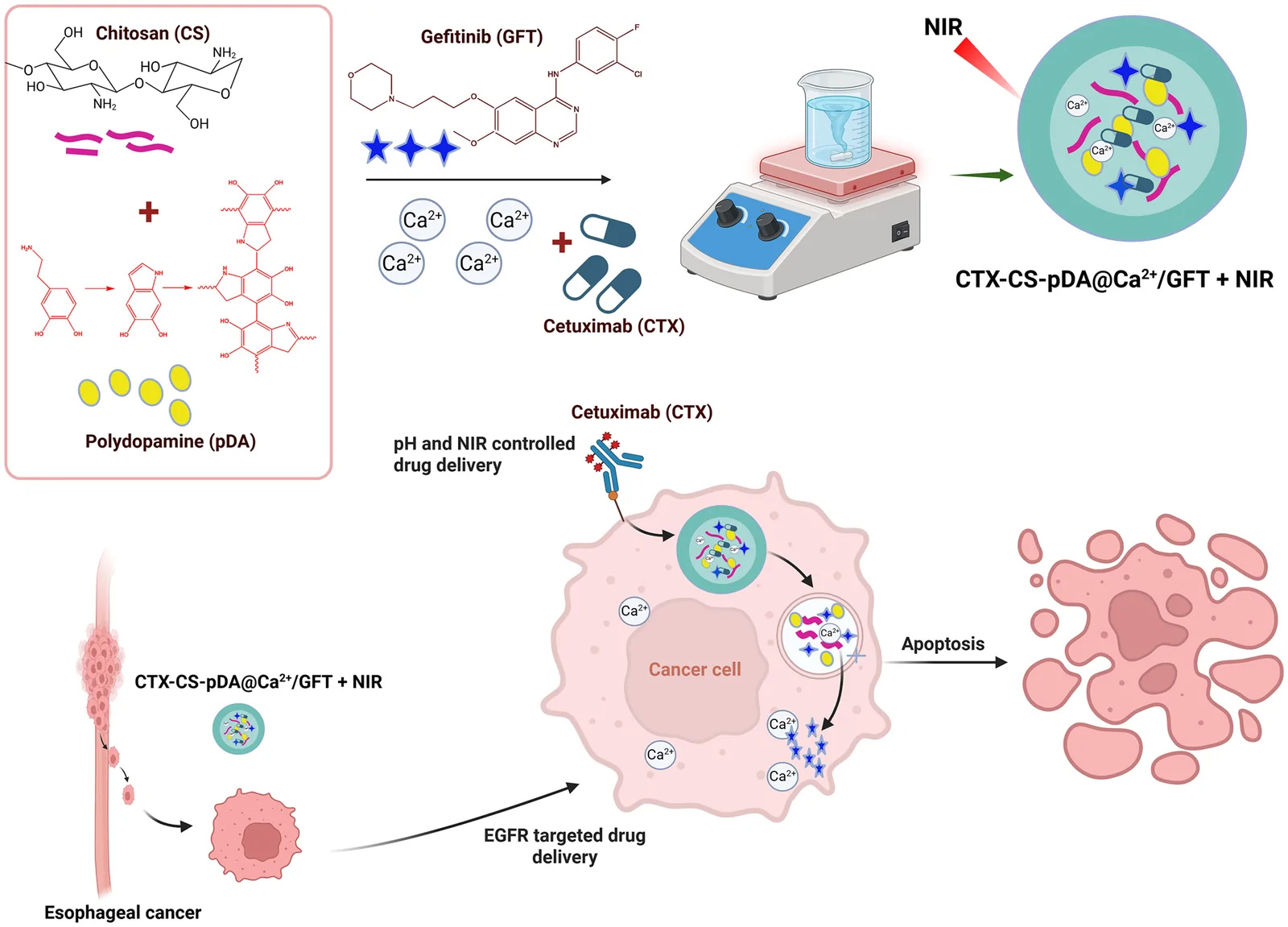

## Introduction

Cancer represents a major global health concern, exerting significant social, medical, and economic burdens. Despite advancements in diagnosis and treatment, the rising prevalence and associated mortality rates continue to challenge healthcare infrastructure and highlight the urgent need for more effective therapeutic strategies. Approximately 16.8% of all fatalities in the globe can be attributed to cancer, and cancer accounts for nearly 22.8% of mortality associated with non-communicable diseases. Cancer accounts for approximately 30.3% of premature deaths among individuals aged 30–69 years and ranks among the top three leading causes of mortality in this age group across 177 of 183 countries [1]. Among these, Esophageal cancer (EC) also presents one of the significant concerns, accounting for 604, 100 new incidences and 544,076 deaths in 2020 worldwide [2]. It ranks as the seventh most common cancer and the sixth leading cause of cancer-related mortality. Although progress has been made in early diagnosis and treatment approaches, the 5-year survival rate for advanced EC remains under 20%, indicating the urgent need for rapid and effective intervention [3]. Additionally, due to the absence of noticeable symptoms in the early stages and the lack of reliable diagnostic methods for early detection, EC is frequently identified only after it has progressed to an advanced stage, with over half of patients already exhibiting metastasis [4]. Even though great advancements have been made regarding the surgical approach and the use of chemotherapy and radiotherapy, mortality rates still remain very high [5]. Survival rates are estimated at around 20% for five years but are below 4% for patients suffering from metastases. For these reasons, there is a great need to find new approaches to therapy for controlling tumor development and metastasis of EC cells. The treatment options for EC include surgery and chemoradiotherapy, which tend to be quite invasive procedures.

The therapeutic antibodies are among the leading biologic products that have been used in treating diseases such as cancer [6]. Although their performance is satisfactory, wider usage has been hindered by the difficulty in the production process due to high costs involved. Cetuximab (CTX) is a chimeric monoclonal antibody that has been used to treat a wide variety of cancers but only through intravenous administration [7]. Nevertheless, its use may bring about side effects such as cardiopulmonary arrest, hypotension, bronchospasm, and cutaneous toxicity [8]. The EGFR is a crucial protein in the process of developing cancers since it enhances the proliferation, migration, and survival of the cells [9]. Therefore, targeted drug therapies are effective in the management of the conditions since the use of EGFR inhibitors such as CTX binds to EGFR and inhibits the enzyme tyrosine kinase [10].

Increased expression of EGFR in numerous preclinical cancer models, including EC, has been shown to lead to increased epithelial cell proliferation, differentiation, and migration [11]. It is important to highlight that overexpression of EGFR has been shown to correlate negatively with the sensitivity of tumors to radiotherapy treatment, leading to decreased radiocurability [12]. Multiple studies have also reported that high EGFR levels correlate with reduced tumor control following radiotherapy [13, 14]. Although recent studies suggest that combining EGFR inhibitors with radiotherapy may improve local tumor control compared to radiotherapy alone, conflicting evidence has also been reported in the literature [15]. Calcium is a vital mineral that plays a fundamental role in both the structural and functional integrity of the human body. It is a key component responsible for the strength and stability of bones and teeth. It is required for the proper functioning of muscles, heart, and nerves [16]. It helps in forming clots and secreting hormones like insulin. Imbalance in calcium levels in the body results in conditions like osteoporosis, arrhythmia, hypertension, and the formation of stones in kidneys [17].

Gefitinib (GFT) is a clinically approved small molecule inhibitor of EGFR that blocks the activity of EGFR by competing with ATP to bind to the kinase domain. Apart from its anticancer properties, the compound has also been shown to have a significant impact on the thickness of the epiphyseal growth plate [18]. In this proposed research work, the use of a NIR- responsive hydrogel of chitosan will be employed for the parenteral administration of GFT in order to increase its effectiveness as a specific EGFR inhibitor. Hydrogels provide biocompatibility, biodegradation, low toxicity, and controlled gelation-sol transition, which facilitates controlled release of the drug without any first-pass effect. The intelligent hydrogel is capable of releasing GFT upon thermal, pH, redox, or pathologically induced changes in the microenvironment.

Hydrogels can be synthesized by natural or synthetic polymers. The main benefits of natural polymers, especially polysaccharides like chitosan (CS) [19], dextran [20], alginate [21], and cellulose [22], are attributed to their great biocompatibility and inherent biological activities. These polymers can be fabricated into hydrogel matrices via physical and chemical crosslinking methods. As a result, natural polymers have shown tremendous promise in biomedical applications such as tissue engineering, wound healing, and drug delivery systems [23]. Out of all these natural polymers, CS, which is obtained from nature, has recently attracted much research interest as an effective carrier material for drug delivery purposes, including the delivery of drugs, peptides, proteins, and even genes [24].

**Fig. 1:**
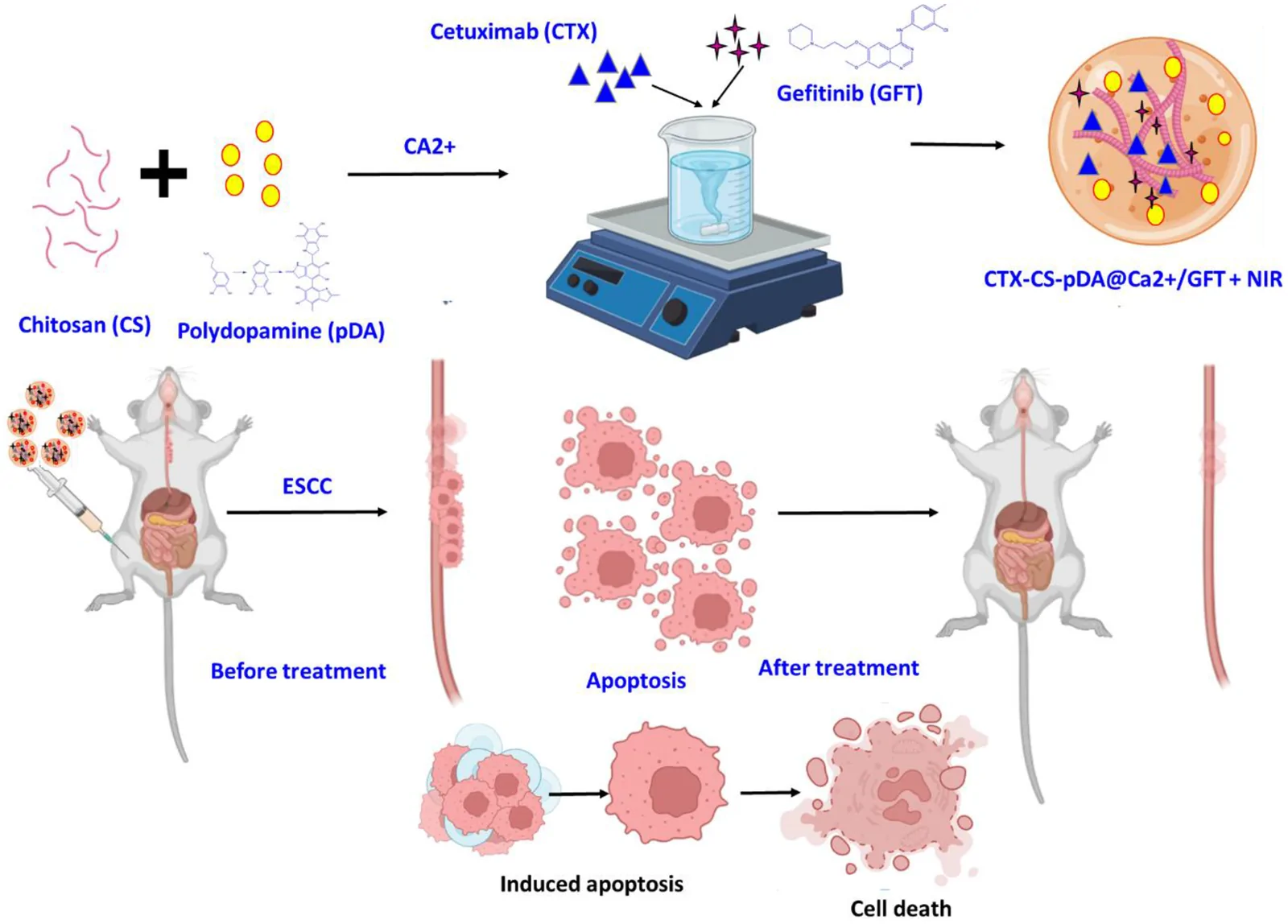
Scheme illustrates the anticancer potential of CTX-CS-pDA/Ca^2+^/GFT + NIR hydrogel on esophageal cancer

Photothermal therapy has recently been established as an efficient method of cancer treatment with limited side effects [25]. Combining photothermal therapy and chemotherapy will increase the efficacy of cancer treatments through synergistic cytotoxicity and will lead to a stronger inhibition of the tumors’ growth. The use of near-infrared (NIR) light irradiation for monitoring temperature variation in the drug carrier system can be the best choice owing to its high penetration and low risk of damaging healthy cells and tissues [26]. Traditional photothermal nanomaterials, such as gold nanoparticles, carbon nanotubes, graphene, and Fe₃O₄ nanoparticle clusters, have been known to show excellence performance in NIR response and photothermal action [27]. Nevertheless, they have problems reaching a well-uniformed distribution in self-healing hydrogels. Furthermore, these materials can potentially cause toxicity issues; metallic nanoparticles are often less biodegradable, while carbon-based materials may induce oxidative stress and inflammatory responses, thereby raising concerns regarding their in vivo safety. In contrast, polydopamine (pDA) has emerged as an efficient photothermal agent due to its excellent biocompatibility, low toxicity, and strong adhesive properties [28]. Hydrogels can be uniformly integrated with pDA nanoparticles, enabling effective photothermal conversion and favorable structural modifications, making them highly suitable for biomedical applications, particularly targeted cancer therapy. In the present study, chitosan was employed as the base polymer to fabricate a chitosan–polydopamine hydrogel, which was further functionalized with cetuximab to enable the co-delivery of Ca^2 +^ and gefitinib for EGFR-targeted therapy of esophageal cancer via Ca^2 +^ -mediated cytotoxic effects and potential immunomodulatory mechanisms. Hence, this study aimed to develop and evaluate a cetuximab (CTX)-functionalized chitosan–polydopamine (CS-pDA) hydrogel co-delivering Ca^2 +^ and gefitinib (GFT), (CTX-CS-pDA@Ca^2+/^GFT) and to investigate its NIR-responsive therapeutic efficacy in promoting Ca^2 +^ -mediated cytotoxicity and cytokine-associated immunomodulatory responses in EC (Fig. 1).

## Materials and methods

### Materials

Chitosan (medium molecular weight, 75–85% deacylated), dopamine hydrochloride, calcium chloride (CaCl₂), gefitinib, EDC·HCl, NHS, cetuximab (CTX), acetic acid, phosphate-buffered saline (PBS), and MTT reagent were purchased from Sigma-Aldrich, St. Louis, MO, USA.

### Preparation of chitosan-polydopamine (CS-PDA) hydrogel

For the preparation of CS-pDA hydrogel, 2% (w/v) CS solution was obtained by dissolving CS in 1% acetic acid with continuous stirring overnight to ensure complete dissolution. Separately, dopamine was dissolved in a Tris-HCl buffer (pH 8.0) to prepare a fresh pDA solution at a concentration of 2 mg/mL, 12 hours before use. The hydrogel was then formulated by mixing the CS and pDA solutions at a volumetric ratio of 2:1. Upon mixing, the pH of the resultant solution was approximately 4.5. To enhance the viscosity of the hydrogel mixture, the pH was gradually adjusted to 6.0 using 2 M NaOH while continuously stirring on a heated plate.

### Loading of calcium (Ca^2+^) and gefitinib (GFT)

To load GFT onto the fabricated hydrogel, 2.0 mg/mL of GFT was initially dissolved in DMSO. Subsequently, 10 mL of CS-pDA hydrogel (0.1 mg/mL) and 20 µL of triethylamine were added dropwise under continuous stirring. To this mixture, 6 mL of PBS (pH 7.4) was introduced, and the mixture was continuously stirred at room temperature until equilibrium was achieved for GFT loading. The resulting GFT loaded CS-pDA hydrogel was centrifuged at 20,000 rpm for 20 minutes to separate unbound components, followed by several washes with distilled water. The characteristic absorbance peak of GFT at 331 nm was used to quantify the unbound drug in the supernatant. Subsequently, cetuximab (CTX) was conjugated onto the GFT-loaded CS-pDA hydrogel through surface interaction/functionalization under gentle stirring conditions, allowing receptor-targeting capability via EGFR binding. The CTX-functionalized hydrogel was then purified by centrifugation and washing to remove excess unbound antibody. After that, the hydrogel solution was gently dropped into a 0.2 M CaCl_2_ solution and allowed to crosslink for 24 hours. Following the crosslinking process, the fabricated hydrogels were thoroughly washed with distilled water multiple times and stored at 4 °C for subsequent experiments.

### Physico-chemical characterization of the fabricated hydrogel

To investigate the functional groups, present in the hydrogel, FT-IR analysis was performed over the spectral range of 400–4000 cm^−1^ using analytical grade KBr pellet preparation. Surface morphology and microstructural features of the hydrogel were visualized through scanning electron microscopy (SEM, JEOL JSM-6490LA). Additionally, dynamic light scattering (DLS) analysis (Brookhaven Instruments Corp., USA) was conducted to assess the hydrogel’s particle size distribution and colloidal stability.

### swelling behavior of CTX-CS-pDA@Ca^2+^/GFT hydrogel

The swelling behavior of the fabricated hydrogel loaded with CTX, Ca^2+^ and GFT was assessed using a method of gravimetric analysis. In brief, the lyophilized hydrogels were initially immersed in PBS (PBS, pH 7.4) at 37 °C to mimic physiological conditions. The initial dry weight was measured prior to immersion. At designated time points, the swollen hydrogels were removed, gently blotted to eliminate surface moisture, and immediately weighed to determine the swollen weight. The swelling ratio (Q) of hydrogel was calculated using the below formula.

Q = (Wswollen - Wdry)/Wdry × 100%.

### Rheological analysis

The rheological characteristics of the CTX-CS-pDA@Ca^2 +^/GFT hydrogel were analysed using a HAAKE™ MARS III rotational rheometer equipped with a 35 mm parallel plate geometry and a fixed gap of 1.0 mm at 37 °C. The hydrogel samples, prepared and stabilized for at least 24 h prior to testing, were carefully placed on the measuring platform. To prevent dehydration and ensure reliable data, a thin layer of silicone oil was applied around the plate edges.

The shear viscosity behavior of the hydrogel was modelled using the Carreau–Yasuda equation, which effectively correlates viscosity (η) with shear rate (γ) and temperature (T) in polymeric systems:

$$\eta \left( {\gamma ,T} \right) = {\eta _0}\left( T \right){\left[ {1 + {{\left( {{{{\eta _0}\left( T \right)\gamma } \over \tau }} \right)}^a}} \right]^{{{n - 1} \over a}}}$$

Where *η* represents shear viscosity (Pa·s), *η₀* is the zero-shear viscosity (Pa·s), *γ* denotes the shear rate (s^− 1^), *τ* is the relaxation time, *a* describes the transition width, and *n* is the flow index indicating shear-thinning behavior. To describe the temperature-dependent viscoelastic response, the Williams–Landel–Ferry (WLF) model was employed for time–temperature superposition analysis. The relationship between the temperature shift factor (*a*_t_) and temperature was expressed as:

$${\rm{log}}\left( {{a_T}} \right) = {{{C_1}\left( {T - {T_0}} \right)} \over {{C_2} + \left( {T - {T_0}} \right)}}$$

where *T₀* is the reference temperature, and *C₁* and *C₂* are empirical constants specific to the hydrogel system. These parameters were determined by nonlinear curve fitting to align with the experimentally derived superposition data.

### In vitro cumulative and NIR-triggered release study

The cumulative release behavior of GFT from the CS-pDA@Ca^2 +^/GFT and CTX-CS-pDA@Ca^2+/^GFT with and without NIR hydrogel was investigated under simulated physiological and tumor-like conditions. A predetermined amount of hydrogel was placed into dialysis membrane bags (molecular weight cutoff: 12–14 kDa) and immersed in 20 mL of phosphate-buffered saline (PBS, pH 1.2, 4.5, 6.5, and 7.4) to mimic physiological conditions and to simulate the acidic tumor microenvironment. The experimental conditions were kept constant at 37 ± 0.5 °C in a shaking incubator at 100 rpm. For each experiment, aliquots of 2 mL were taken out at certain intervals (1, 2, 4, 8, 12, 24, 48, and 72 h). These 2 mL were then refreshed with an equal amount of buffer solution to keep the system in a sink state. The percentage release of GFT from the samples was measured by the UV-visible spectrophotometric method at λ_max = 340 nm using the already established calibration curve.

For NIR-triggered release evaluation, hydrogel samples were irradiated with near-infrared (NIR) light (808 nm, 1.5 W·cm^− 2^) for 5 min at predetermined intervals to induce photothermal activation. The surface temperature of the samples during irradiation was monitored using a thermocouple or IR thermometer. Control groups were maintained under identical conditions without NIR exposure. The effect of NIR irradiation on the release behavior was analyzed by comparing cumulative release profiles obtained under both irradiated and non-irradiated conditions. Finally, the release kinetics were modelled using zero-order, first-order, Higuchi, and Korsmeyer–Peppas equations to elucidate the dominant mechanism governing GFT release from the hydrogel matrix.

### In vitro studies

#### Cell culture and maintenance

The human esophageal squamous cell carcinoma cell line KYSE-140 cells (human esophageal squamous carcinoma cell line; RRID: CVCL_1348) were obtained from the Cell Bank of the Chinese Academy of Sciences (Shanghai, China). Cells were cultured in Dulbecco’s Modified Eagle Medium (DMEM) supplemented with 10% fetal bovine serum (FBS), 1% penicillin-streptomycin, and 1% L-glutamine. The cells were grown under humidified conditions in an incubator at 37 °C and a 5% CO₂ atmosphere. Cultured media were changed every two to three days for maintenance of suitable cell growth conditions. After reaching around 80–90% confluency, the cells were detached by using a 0.25% trypsin-EDTA solution and then reseeded into the fresh dishes at a proper split ratio. Cells were harvested in their logarithmic phase of growth for all experiments.

#### Cell viability assay

Cell viability was assessed through the CCK-8 assay method. Specifically, the KYSE-140 EC cells were seeded in 96-well plates at a cell density of 2 × 10^4^ cells per well and left to settle for 24 hours under culture conditions of 37 °C and 5% CO₂. The treatments were done in a similar 96-well plate setup. To determine the optimal treatment concentration, a dose–response study was performed by exposing KYSE-140 cells to varying concentrations (0–100 µg/mL) of GFT, CS-pDA, CS-pDA@Ca^2 +^/GFT, and CTX-CS-pDA@Ca^2 +^/GFT for 24 h. From the results obtained, a concentration of 50 µg/mL was determined to be ideal for further analysis due to its ability to significantly reduce cell viability without being too cytotoxic. Further studies involved the treatment of cells with GFT, Ca^2 +^, GFT + Ca^2 +^, NIR only, CS-pDA, CS-pDA@Ca^2 +^/GFT, CTX-CS-pDA@Ca^2 +^/GFT, and CTX-CS-pDA@Ca^2 +^/GFT + NIR at the final concentration of 50 μg/mL (drug/hydrogel equivalent) for 24 h. The concentration of Ca^2 +^ ions in all treatment groups was maintained at 1 mM, based on previously reported cytocompatibility tests [29]. In order to study the effect of NIR exposure, the samples were exposed to a near-infrared (NIR) laser with a wavelength of 808 nm, power density of 1.5 W/cm^2^, and irradiation time of 5 min after applying treatments [30]. To analyze the effect of tested substances, 10 µL of CCK-8 solution was added to each well and then incubated in darkness at 37 °C for another 2 h. Cell viability was estimated by measuring absorbance at 450 nm on a microplate reader (PerkinElmer, USA).

#### Cell proliferation assay

Cell proliferation was assessed by the use of the Cell-Light EDU Apollo 488 In Vitro Imaging Kit (RiboBio). EDU is a thymidine analog that gets integrated into newly made DNA in the S phase, allowing the detection of cells that are dividing. Specifically, KYSE-140 esophageal cancer cells were cultured in a 96-well plate at the density of 2 × 10^4^ cells/well and grown for 24 h in normal cell culture conditions (37 °C, 5% CO₂). All experiments were conducted in a 96-well plate. The cells were treated with GFT, CS-pDA, CS-pDA@Ca^2 +^/GFT, CTX-CS-pDA@Ca^2 +^/GFT, and CTX-CS-pDA@Ca^2 +^/GFT+NIR with the final drug concentration of 50 µg/mL for 24 h. For the NIR group, near-infrared irradiation (λ = 808 nm, power density = 1.5 W/cm^2^, time = 5 min) was delivered to the wells after treatment. After treatment, the culture medium was replaced with fresh medium containing 10 µM EDU, and the cells were further incubated for 24 h. Subsequently, cells were fixed, treated with glycine, and stained according to the manufacturer’s protocol. Hoechst 33,342 was used for nuclear counterstaining. The proportion of EDU-positive cells, representing DNA synthesis and cell proliferation, was visualized and quantified using a fluorescence microscope (Olympus IX73; Olympus Corp, Tokyo, Japan).

#### Morphological assessment of cells following hydrogel exposure

To assess the anti-proliferative effect and cytotoxic response of the fabricated hydrogels, KYSE-140 EC cells were subjected to treatment with NIR only, Ca^2+^, GFT, GFT + Ca^2+^, CS-pDA, CS-pDA@Ca^2+^/GFT, CTX-CS-pDA@Ca^2+^/GFT and CTX-CS-pDA@Ca2+/GFT + NIR. For AO/EB (Acridine Orange/Ethidium Bromide) staining, cells (2 × 10^4^ cells/mL) were seeded into 96-well plates and incubated overnight to allow adherence. Cells were detached after 24 hours of treatment with 20 µL of trypsin solution per well, washed twice with PBS, and then stained with AO/EB (100 μg/mL) by using 4 µL of the dye solution. Stained cells were transferred to glass slides and observed using a fluorescence microscope. Live cells were stained with green color, while apoptotic or dead cells were stained with red color due to the accumulation of AO and EB in their nuclei, respectively.

To study the nuclear structure, KYSE-140 cells (1 × 10^5^ cells/well) were placed in 48-well plates having DMEM and treated with NIR only, Ca^2+^, GFT, GFT + Ca^2+^, CS-pDA, CS-pDA@Ca^2+^/GFT, CTX-CS-pDA@Ca^2+^/GFT and CTX-CS-pDA@Ca2+/GFT + NIR. Cells were kept for 24 hours in an incubator at 37 °C, and then fixed with methanol and stained with DAPI (1 µg/mL) for 10 minutes. Excess dye was removed by washing with PBS, and fluorescence images were captured using a fluorescence microscope to observe chromatin condensation and nuclear fragmentation, characteristic of apoptosis.

#### Mitochondrial membrane potential (ΔΨm) analysis using JC-1 staining

Mitochondrial membrane potential was determined by using the JC-1 fluorescence dye, wherein healthy mitochondria fluoresce and appears in red color and damaged mitochondria (depolarized) show green fluorescence. In brief, KYSE-140 cells were cultured in 6-well plates and incubated with NIR only, Ca^2+^, GFT, GFT + Ca^2+^, CS-pDA, CS-pDA@Ca^2+^/GFT, CTX-CS-pDA@Ca^2+^/GFT and CTX-CS-pDA@Ca2+/GFT + NIR hydrogel for 12 hours. Cells were stained with JC-1 (2 μg/mL) in dark conditions for 30 minutes at 37 °C. Later on, the cells were washed with PBS buffer and trypsinized. After that, the cell suspension was obtained through centrifugation. A fluorescence signal was observed under the fluorescence microscope having a UV filter with an excitation wavelength of 450–490 nm. Red and green fluorescence intensities were recorded to evaluate mitochondrial integrity and depolarization.

#### Evaluation of cell migration and cell invasion

A wound healing assay was carried out to assess the inhibitory effect of the fabricated hydrogel on the migratory ability of KYSE-140 cells. For the analysis, the cells were seeded in 6-well plates at a density of 1 × 10^6^ cells per well and cultured until a uniform monolayer was achieved. A linear scratch was carefully created using a sterile micropipette tip, and detached cells were removed by washing with PBS. The cells were then treated with NIR only, Ca^2+^, GFT, GFT + Ca^2+^, CS-pDA, CS-pDA@Ca^2+^/GFT, CTX-CS-pDA@Ca^2+^/GFT and CTX-CS-pDA@Ca2+/GFT + NIR while untreated cells served as control. Following 0 hours and 24 hours of incubation, the wound area was imaged under an inverted microscope to assess cell migration and wound closure percentage compared to the control group.

To evaluate the effect of fabricated hydrogel on cancer cell invasiveness, KYSE-140 cells were treated with NIR only, Ca^2+^, GFT, GFT + Ca^2+^, CS-pDA, CS-pDA@Ca^2+^/GFT, CTX-CS-pDA@Ca^2+^/GFT and CTX-CS-pDA@Ca2+/GFT + NIR for 24 hours. Following treatment, cells were subjected to trypsinization, resuspended, and 100 µL of cell suspension (1 × 10^4^ cells) was added to the upper chamber of a Transwell insert (8 µm pore size, Corning), which had been pre-coated with Matrigel (BD Biosciences) to stimulate the extracellular matrix. After 24 h of incubation, non-invading cells remaining on the upper side of the lower surface were fixed with 4% paraformaldehyde for 15 min and stained with 0.1% crystal violet for 5 minutes. After that, invaded cells were visualized under an inverted phase contrast microscope (Olympus IX73; Olympus Corp., Tokyo, Japan) and quantification was performed by counting cells in eight randomly selected fields per insert.

#### Intracellular ROS determination

The intracellular reactive oxygen species (ROS) levels were quantified to examine oxidative stress induced by hydrogel treatment with and without NIR. Briefly, KYSE-140 cells were exposed to NIR alone, Ca^2+^, GFT, GFT + Ca^2+^, CS-pDA, CS-pDA@Ca^2+^/GFT, CTX-CS-pDA@Ca^2+^/GFT and CTX-CS-pDA@Ca2+/GFT + NIR for 24 hours. After treatment, the cells were washed with PBS and incubated with 10 μM 2′,7′-dichlorofluorescein diacetate (DCFH-DA; Sigma-Aldrich, St. Louis, MO, USA) in serum-free medium at 37 °C for 30 minutes in the dark. Subsequently, the cells were trypsinized, resuspended in PBS, and examined under a fluorescence microscope (Olympus IX73; Olympus Corp., Tokyo, Japan) at an excitation wavelength of 488 nm and emission wavelength of 525 nm. The intensity of green fluorescence corresponded to the intracellular ROS levels, allowing comparison between treated and control groups.

#### Intracellular measurement of Ca^2+^ using Fluo-4 AM assay

Intracellular Ca^2 +^ production in KYSE-140 cells after treatment with NIR only, Ca^2+^, GFT, GFT + Ca^2+^, CS-pDA, CS-pDA@Ca^2+^/GFT, CTX-CS-pDA@Ca^2+^/GFT and CTX-CS-pDA@Ca2+/GFT + NIR was determined through the Fluo-4 Am assay. In short, the cells were seeded in 96-well plates at a density of 2 × 10^4^ cells per well and allowed to adhere overnight under standard culture conditions. The cells were then treated according to the experimental groups and incubated for 24 h. Following treatment, intracellular Ca^2 +^ levels were determined using the calcium-sensitive fluorescent probe Fluo-4 AM. Briefly, cells were incubated with Fluo-4 AM (5 µM) in serum-free medium at 37 °C for 30 min in the dark. After incubation, the cells were gently washed with PBS to remove excess dye. For the NIR-treated group, cells were exposed to an 808 nm near-infrared laser at a power density of 1.5 W/cm^2^ for 5 min prior to staining. Fluorescence images were captured using a fluorescence microscope, and the intensity of green fluorescence was quantified using ImageJ software. The fluorescence intensity is directly proportional to intracellular Ca^2 +^ levels.

#### Flowcytometry analysis

Apoptotic cell death was quantitatively assessed using Annexin V-FITC/propidium iodide (PI) dual staining followed by flow cytometric analysis. Briefly, KYSE-140 cells were treated with NIR only, Ca^2+^, GFT, GFT + Ca^2+^, CS-pDA, CS-pDA@Ca^2+^/GFT, CTX-CS-pDA@Ca^2+^/GFT and CTX-CS-pDA@Ca2+/GFT + NIR hydrogel for 24 h. After treatment, cells were harvested, washed twice with cold PBS, and resuspended in binding buffer. Subsequently, the cells were stained using 5 µL Annexin V-FITC and 5 µL propidium iodide (PI) following the manufacturer’s protocol and incubated at room temperature for 15 min. The stained cells were subjected to flow cytometric analysis using the BD FACSCanto II flow cytometer from BD Biosciences (San Jose, CA, USA). Annexin V-FITC and PI fluorescent signals were measured in the FITC and PI channels, respectively (FITC: Excitation/Emission = 488/530 nm; PI: Excitation/Emission = 488/585 nm). Four different cell populations based on fluorescence signal intensity were distinguished, which included viable cells (Annexin V^−^/PI^−^), early apoptotic cells (Annexin V^+^/PI^−^), late apoptotic cells (Annexin V^+^/PI^+^), and necrotic cells (Annexin V^−^/PI^+^).

### In vivo animal studies

#### Animal grouping and treatment

Albino mice (5–6 weeks old) were maintained under specific pathogen-free (SPF) conditions with a 12 h light/dark cycle and provided with ad libitum access to food and water. All animal experiments were conducted in accordance with institutional guidelines for the care and use of laboratory animals and complied with the ARRIVE guidelines. The experimental protocol was reviewed and approved by the Experimental Animal Ethics Committee of Beijing Jinglai Huake Biotechnology Co., Ltd(NO. JLSW-2023117).

Animals were acclimatized for one week prior to experimentation. Healthy mice with an average body weight of 18–22 g were used in this study. For tumor induction, 5 × 10^6^ KYSE-140 cells were suspended in 200 µL of PBS and subcutaneously injected into the upper right flank of each animal. Mice bearing tumors of approximately 5–6 mm in diameter were randomly allocated into different treatment groups to minimize selection bias. Each group consisted of six animals. The groups included Group I (sham control, normal saline); Group II (free GFT); Group III (CS-pDA); Group IV (CTX-CS-pDA hydrogel); Group V (CTX-CS-pDA@Ca^2 +^/GFT); and Group VI (CTX-CS-pDA@Ca^2 +^/GFT + NIR). Additional control conditions (NIR-only, Ca^2 +^, and Ca^2 +^ + GFT) were evaluated in vitro; however, these groups were not included in the in vivo study, as the experimental design was focused on assessing the combined therapeutic efficacy of the hydrogel system while minimizing unnecessary animal usage in accordance with ethical guidelines. All formulations were applied to mice by intratumoral administration once every three days for four cycles of treatments. The dosing strategy for in vivo experiments was established based on prior studies and the localized nature of the hydrogel delivery system. The CTX-CS-pDA@Ca^2 +^/GFT hydrogel was administered via intratumoral injection at a concentration of 2 mg/mL with an injection volume of 100 µL per tumor (equivalent to ~ 10 mg/kg body weight, assuming an average mouse weight of 20 g). The formulation was loaded with GFT at an equivalent dose of approximately 2 mg/kg [31], CTX at ~ 1 mg/kg (≈20 µg per mouse) [32], and Ca^2 +^ at a localized concentration of ~ 2 mM to induce Ca^2 +^ -mediated apoptotic signaling. NIR irradiation (808 nm, 0.8 W/cm^2^ for 5–10 min) was applied to activate the photothermal properties of the hydrogel and enhance therapeutic efficacy. This dosing approach enables sustained and localized drug release, ensuring effective tumor targeting while minimizing systemic toxicity. In order to reduce any bias and achieve a more objective approach in terms of scientific design, tumor volume and histopathology analysis were done by blinded observers. No animals or data points were excluded from the analysis.

#### In vivo xenograft study

Tumor volume and body weight were recorded every three days. Tumor volume (in mm^3^) was calculated using the formula: 0.5 ×D x d^2^, where D is the longest diameter and d is the shortest diameter. After 13 days of treatment, all mice were euthanized using an approved humane procedure. Blood samples were collected to assess serum glutamic oxaloacetic transaminase (SGOT), serum urea, serum creatinine, and serum alanine aminotransferase (ALT) levels for systemic toxicity evaluation.

#### Histopathology analysis

Tumor tissues and major organs, including the esophagus, kidney, liver, lungs and spleen were carefully excised from the experimental animals and fixed in 10% neutral buffered formalin overnight to preserve their structural integrity. Following fixation, the specimens were processed for paraffin embedding, trimmed, and sectioned longitudinally into 5-µm slices using a microtome. Deparaffination of the ribbon tissues was carried out using xylene, followed by gradual rehydration through a gradient series of ethanol. Staining for the histological procedure was done by immersing the section in hematoxylin for 3 minutes, followed by rapid differentiation in 1:7 hydrochloric acid/ethanol for 10 seconds. The slides were then counterstained with eosin for 10 minutes, dehydrated in ethanol, cleared in xylene, and mounted using neutral balsam. The prepared slides were examined under an inverted microscope to evaluate microscopic alterations, cellular morphology, tissue integrity, and treatment-associated changes in response to GFT, CS-pDA, CS-pDA@Ca^2+^/GFT, CTX-CS-pDA@Ca^2 +^/GFT and CTX-CS-pDA@Ca^2 +^/GFT + NIR hydrogel formulations.

#### Quantitative real-time PCR and Western blot analysis

In the in vivo study, the expression levels of EGFR-targeted signaling and apoptosis-related genes and proteins were analyzed using quantitative real-time PCR (qRT-PCR) and Western blotting to elucidate the molecular mechanisms underlying the therapeutic effect of fabricated hydrogels. For the analysis, total RNA was extracted from tumor tissue samples using TRIzol reagent (Invitrogen, USA) following the manufacturer’s protocol, and RNA purity and concentration were assessed using a NanoDrop spectrophotometer. The synthesis of complementary DNA (cDNA) was achieved from 1 µg of total RNA by means of reverse transcription using the reverse transcription kit (Thermo Fisher Scientific, USA). Relative mRNA expression analysis of the genes for EGFR, AKT, PTEN, Bcl-2, Bax, caspase-3, and caspase-9, along with pro-inflammatory cytokines such as IL-6, TNF-α, and IFN-γ, was performed using qRT-PCR with SYBR Green Master Mix (Applied Biosystems, USA) on the StepOnePlus Real-Time PCR system (Applied Biosystems, USA) with GAPDH as the housekeeping gene. The relative fold change in gene expression was calculated using the 2^−ΔΔCt method.

For the study of protein expression, tumors were ground in RIPA lysis buffer containing protease and phosphatase inhibitors and centrifuged to get the supernatant. The protein concentration was measured by a BCA protein assay kit (Thermo Fisher Scientific, USA). Equivalent amounts of proteins (30–50 µg) were fractionated using SDS-PAGE and then electrotransferred to PVDF membranes. The membranes were blocked with 5% non-fat milk and incubated overnight at 4 °C with the following primary antibodies (Cell Signaling Technology, Danvers, MA, USA) such as anti-EGFR (Cat# 2646, RRID: AB_2230881), anti-phospho-AKT (Ser473) (Cat# 4060, RRID: AB_2315049), anti-AKT (Cat# 9272, RRID: AB_329827), anti-phospho-PTEN (Ser380/Thr382/383) (Cat# 9554, RRID: AB_10694028), anti-PTEN (Cat# 9559, RRID: AB_390810), anti-Bcl-2 (Cat# 3498, RRID: AB_1903909), anti-Bax (Cat# 2772, RRID: AB_10695870), caspase-3 (total) (Cat# 9662, RRID: AB_331439), cleaved caspase-3 (Cat# 9661, RRID: AB_2341188), caspase-9 (total) (Cat# 9508, RRID: AB_2068621), and cleaved caspase-9 (Cat# 9505, RRID: AB_2068623) at a dilution of 1:1000. After washing, membranes were incubated with HRP-conjugated anti-rabbit IgG secondary antibody (Cell Signaling Technology, Danvers, MA, USA; Cat# 7074, RRID: AB_2099233) at a dilution of 1:5000 for 1 h at room temperature. Immunoreactive bands were visualized using an enhanced chemiluminescence (ECL) detection system. The relative protein expression was normalized to β-actin (mouse monoclonal anti-β-actin antibody; Cat# 3700, RRID: AB_2242334) and quantified using ImageJ software.

### Statistical analysis

All experiments were performed in triplicate, and results are expressed as mean ± SD. Statistical analysis was carried out using one-way ANOVA followed by Tukey’s post hoc test with GraphPad Prism software, and exact *p*-values are presented in the figures. The sample size (*n* = 6) for in vivo studies was selected based on prior xenograft experiments to ensure sufficient statistical power for tumor growth inhibition analysis.

## Results and discussion

### Physicochemical characterization of fabricated CTX-CS-pDA@Ca_2_^+^/GFT hydrogel

**Fig. 2:**
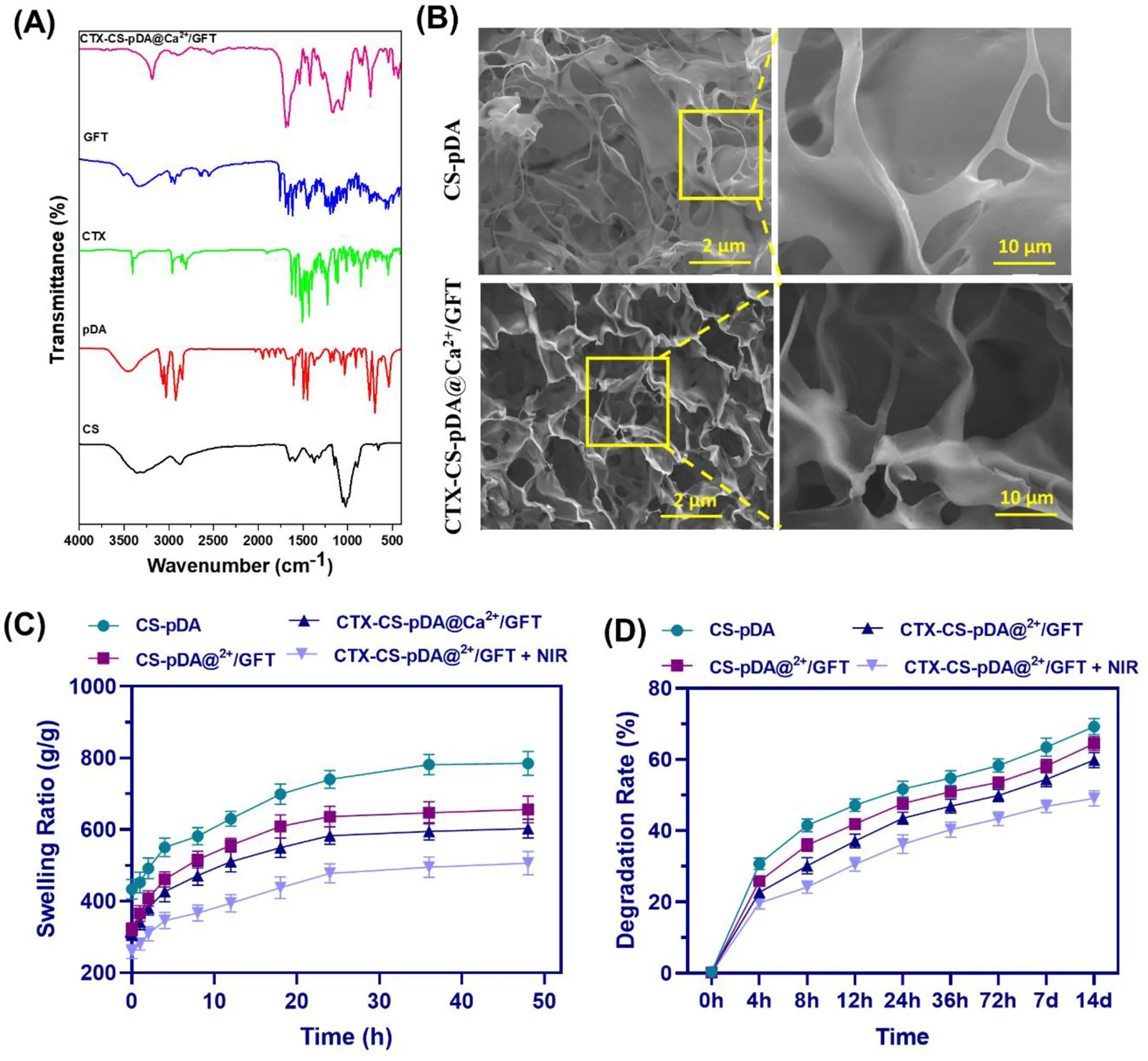
(**A**) FTIR spectra of CS, pDA, CTX, GFT, and CTX-CS-pDA@Ca^2 +^/GFT hydrogel showing characteristic functional group peaks and successful material integration;(**B**) SEM analysis displaying the porous architecture of CS-pDA and CTX-CS-pDA@Ca^2 +^/GFT hydrogels at different magnifications; (**C**) Swelling profiles of various hydrogel formulations over time; (**D**) Degradation behavior of the hydrogels tracked up to 14 days, demonstrating controlled and formulation-dependent mass loss

FTIR analysis was used to check for the successful loading of CS, pDA, CTX, and GFT in the CTX-CS-pDA@Ca^2 +^/GFT hydrogel system (Fig. 2A). It can be seen that the FTIR spectrum of CS contained the specific absorption peak of the amide I group at 1650 cm^−1^, the absorption band of the amide II group at 1560 cm^−1^, and the wide O-H/N-H stretching vibration band from 3200 to 3500 cm^−1^. GFT showed strong absorption peaks attributed to C–F stretching and aromatic ring vibrations between 1000 and 1300 cm^−1^, and a distinct N-H stretching shoulder at approximately 3300 cm^−1^. Furthermore, CTX presented broad protein-associated peaks, including amide I (~1655 cm^−1^) and amide II (~1540 cm^−1^), which are consistent with its antibody structure. In the composite CTX-CS-pDA@Ca^2 +^/GFT hydrogel spectrum, the broad peak obtained at 3200–3500 cm^−1^ corresponds to O-H/N-H vibrations, which indicates hydrogen bonding between CS, pDA, and CTX. In addition, the amide I/II absorption peak raised within the range of 154–1600 cm^−1^ reveals protein polymer interactions. The aromatic region raised in the range of 1450–1600 cm^−1^ exhibited combined features of pDA and GFT, verifying drug incorporation. Additionally, GFT-specific peaks between 1050 and 1250 cm^−1^ were also seen but at low intensities, indicating the embedding of GFT molecules into the hydrogel system (Fig. 2A). Therefore, the peak fusion as well as a little variation in the IR spectra could be indicative of successful integration and intermolecular interactions of CS, pDA, CTX, Ca^2 +^ cross-linking, and GFT in the hydrogel formulation.

SEM images showed that both CS-pDA and CTX-CS-pDA@Ca^2 +^/GFT hydrogels had an extremely porous and sponge-like structure (Fig. 2B). In comparison to the CS-pDA system alone, the presence of drugs and Ca^2 +^ ions in the hydrogel matrix resulted in thickened pores and narrower internal channels of the hydrogel system. Further, both hydrogel systems revealed a gradual increase in the degree of swelling with time. The CS-pDA hydrogel had the highest degree of swelling, whereas Ca^2 +^ crosslinked and drug-laden hydrogels possessed a lower but steady hydration effect owing to the denser cross-linking (Fig. 2C). It is important to note that when the hydrogel system was exposed to NIR, the degree of swelling became even less in the case of CTX-CS-pDA@Ca^2 +^/GFT. This was indicative of further compactification of the hydrogel matrix, which occurred because of photothermal effects. Continuous degradation was observed for all the hydrogel matrices over 14 days (Fig. 2D). The CS-pDA hydrogel matrix degraded at the highest rate compared to Ca^2 +^ cross-linked and drug-loaded hydrogels, indicating better structural stability.

### Rheological analysis

**Fig. 3:**
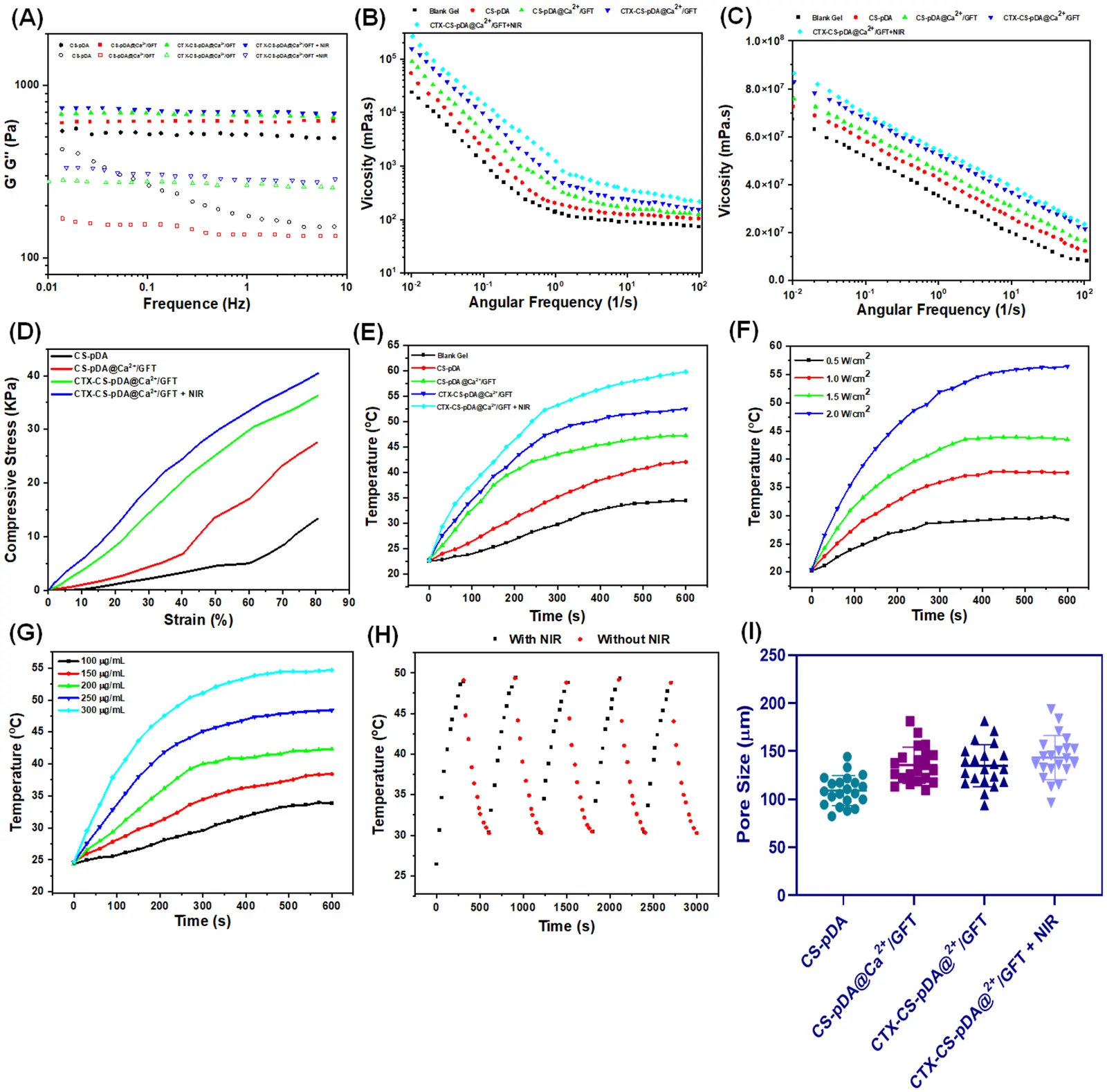
Rheological, mechanical, photothermal, and structural characterization of hydrogel formulations. (**A**) Frequency-dependent storage (G′) and loss (G″) moduli of different hydrogels; (**B-C**) Viscosity profiles as a function of angular frequency demonstrating shear-thinning behavior; (**D**) Compressive stress–strain curves showing Mechanical reinforcement after Ca^2 +^ crosslinking, drug loading, and NIR exposure; (**E**) Photothermal heating curves of hydrogels under NIR irradiation; (**F**) Temperature elevation at different laser power densities;(**G**) Concentration-dependent photothermal response; (**H**) Repeatable heating–cooling cycles confirming photothermal stability; (**I**) Pore size distribution comparison among hydrogel formulations

Frequency sweep test results showed that for all types of hydrogels, G′ values were significantly greater than G″, suggesting an overall elastic nature (Fig. 3A). The CTX-CS-pDA@Ca^2 +^/GFT hydrogel had a higher storage modulus, which suggested that the hydrogel was more rigid due to the presence of ionic bonds and incorporation of drugs. Shear thinning was observed in viscosity studies where the viscosity values decreased with increasing angular frequency (Fig. 3B & C). Among them, CTX-CS-pDA@Ca^2 +^/GFT and its NIR-irradiated counterpart maintained higher viscosities throughout the testing range, reflecting an improved internal network structure. Uniaxial compression testing showed a steady rise in compressive stress with increasing strain. CTX-CS-pDA@Ca^2 +^/GFT + NIR presented the highest compressive strength, surpassing CS-pDA and CS-pDA@Ca^2 +^/GFT (Fig. 3D). The additional reinforcement under NIR irradiation suggests heat-induced tightening of polymer chains that improves load-bearing capacity.

### NIR light-responsive assay

Upon exposure to NIR light, the temperature of the hydrogel increased progressively, with CTX-CS-pDA@Ca^2 +^/GFT + NIR showing the most pronounced photothermal elevation, followed by CS-pDA@Ca^2 +^/GFT and CS-pDA (Fig. 3E). Increasing laser power densities produced a dose-dependent rise in temperature, with 2.0 W/cm^2^ achieving the steepest heating curve (Fig. 3F). Similarly, escalating the hydrogel concentration resulted in higher photothermal output, confirming concentration-dependent heat generation (Fig. 3G). The results from cyclic irradiation showed reliable thermal heating-cooling cycles, reflecting very good photothermal stability without loss of signal (Fig. 3H). Additionally, pore analysis results indicated structural differences between samples. In the case of CS-pDA, there were small and consistent pores. However, upon crosslinking with Ca^2 +^ and incorporation of drugs, the size of the pores was slightly increased. The sample of CTX-CS-pDA@Ca^2 +^/GFT, particularly after NIR irradiation, had relatively larger pores (Fig. 3I).

### Drug loading, entrapment efficiency, drug release kinetics

**Fig. 4:**
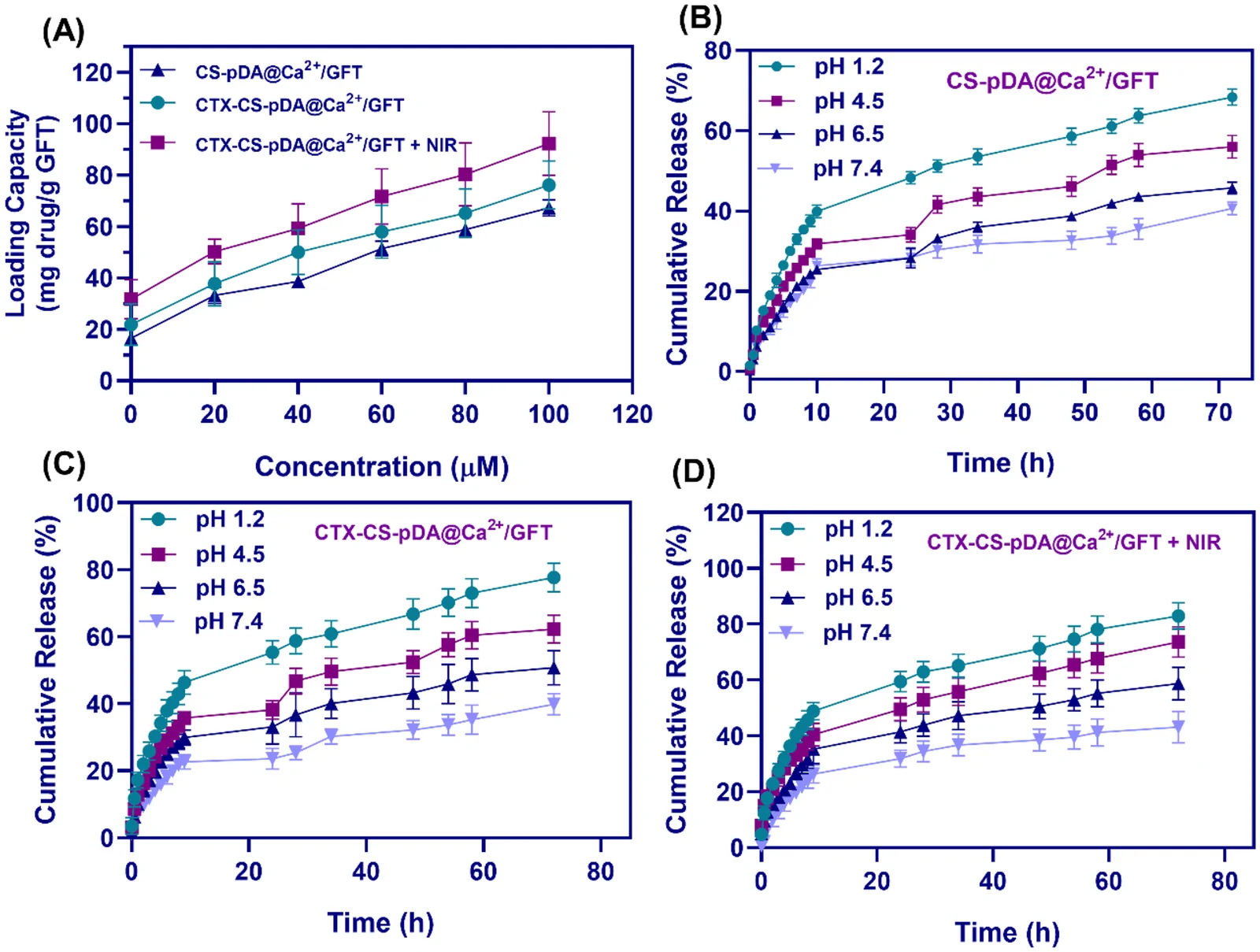
(**A**) Drug loading capacity of CS-pDA@Ca^2 +^/GFT, CTX-CS-pDA@Ca^2 +^/GFT, and CTX-CS-pDA@Ca^2 +^/GFT + NIR hydrogels. (**B–D**) pH-responsive and NIR-triggered in vitro release profiles of GFT from CS-pDA@Ca^2 +^/GFT, CTX-CS-pDA@Ca^2 +^/GFT, and CTX-CS-pDA@Ca^2 +^/GFT + NIR hydrogels at 37 °C under different pH conditions (pH 1.2, 4.5, 6.5, and 7.4). Data are presented as mean ± SD (*n* = 3). Statistical significance was analyzed using one-way ANOVA followed by Tukey’s multiple comparison test (*p < 0.05, **p < 0.01, ***p < 0.001)

The loading efficiency of drugs in the CS-pDA@Ca^2 +^/GFT, CTX-CS-pDA@Ca^2 +^/GFT, and CTX-CS-pDA@Ca^2 +^/GFT + NIR systems followed a concentration dependence, suggesting a proportional increase in the number of sites for active association and drug encapsulation in response to increasing concentrations. Significantly, in response to NIR light, the CTX-CS-pDA@Ca^2 +^/GFT system was able to exhibit significantly higher drug loading efficiency than its counterpart without exposure to NIR light. Such an increase can be explained by the presence of a photothermal effect triggered by the pDA matrix upon NIR illumination, leading to the formation of a more open hydrogel structure and improved penetration of the drug (Fig. 4A). The maximum loading capacity was evaluated by fitting the data to the Langmuir adsorption model, confirming the hydrogel’s strong affinity toward GFT molecules (Table 1).

**Table 1:** Association constants, static quenching constants, loading capacity and entrapment efficiency of GFT in CS-pDA@Ca^2+^/GFT, CTX-CS-pDA@Ca^2+^/GFT and CTX-CS-pDA@Ca^2+^/GFT + NIR

Formulation	**K**_**sv**_ **± SE (10**^**4**^ **M**^**−1**^)	**V ± SE (10**^**4**^ **M**^**−1**^)	**L**_**max**_ **± SE (mg/drug GFT)**	EE (%) ± SE
CS-pDA@Ca^2+^/GFT	1.35 ± 0.06^a^	0.49 ± 0.04^a^	153 ± 12^a^	74.6 ± 2.4
CTX- CS-pDA@Ca^2+^/GFT	1.48 ± 0.15^ab^	0.54 ± 0.0^ab^	168 ± 11^b^	81.3 ± 3.2
CTX- CS-pDA@Ca^2+^/GFT + NIR	1.63 ± 0.16^b^	0.56 ± 0.04^b^	185 ± 11^c^	86.4 ± 3.5

Superscript letters (^a^, ^b^, ^c^) indicate statistical grouping based on significance (*p* ≤ 0.05)

The pH-dependent and NIR-responsive release behavior of GFT from CS-pDA@Ca^2 +^/GFT, CTX-CS-pDA@Ca^2 +^/GFT, and irradiated CTX-CS-pDA@Ca^2 +^/GFT hydrogels was investigated at 37 °C by changing pH levels to 1.2, 4.5, 6.5, and 7.4 (Fig. 4B–D). All systems showed a pH-responsive release profile, where low pH values promoted the diffusion of GFT from the polymer matrix. At pH 7.4, corresponding to a physiological pH level, both CS-pDA@Ca^2 +^/GFT and CTX-CS-pDA@Ca^2 +^/GFT showed slow release with a release ratio close to 25–30%. A gradual increase in cumulative release was observed when the pH was reduced to 6.5, which mimicked the tumor microenvironment, resulting in 40–45% release for CS-pDA@Ca^2 +^/GFT and 35–40% release for CTX-CS-pDA@Ca^2 +^/GFT because of slight swelling and partial protonation of CS molecules. A greater extent of release of GFT was observed at a pH value of 4.5 due to swelling, wherein the total release was between 50 and 55%. The maximum release occurred at pH 1.2 in 24 hours at a rate of 60–70%, which suggests improved hydrolytic relaxation of the matrix.

The response of the drug release from CTX-CS-pDA@Ca^2 +^/GFT was significantly enhanced upon irradiation with 808 nm NIR light (1.5 W/cm^2^; irradiation for 5 min at a time). The accumulated amount of released GFT was found to be 55, 78, 85, and 90% at pH values of 7.4, 6.5, 4.5, and 1.2, respectively (Fig. 4D). The remarkable improvement in the response can be associated to the localized heat produced via the photo-to-thermal conversion effect of the pDA moiety, which induces temporary swelling of the hydrogel network and enhances chain mobility, thus promoting drug diffusion. These findings provide conclusive evidence for the pH-sensitive and photothermally responsive drug release characteristics of CTX-CS-pDA@Ca^2 +^/GFT. GFT’s absorbance-dependent quantification showed that the drug delivery from the hydrogel upon NIR irradiation was characterized by a steeper release profile than the control, indicating a light-induced release approach [33]. The thermal response of the CTX-CS-pDA system, enabled by the cross-linking of Ca^2 +^, facilitates the structural change of the hydrogel due to NIR irradiation, resulting in faster diffusion of drugs [34, 35].

Analysis of the kinetic model of the drug-release profile revealed that the drug release kinetics of GFT from CTX-CS-pDA@Ca^2 +^/GFT hydrogel was better represented by Higuchi’s equation, where the correlation factor was higher (R^2^ = 0.97), indicating that diffusion is the predominant drug-releasing mechanism. This claim was additionally confirmed by Korsmeyer-Peppas analysis, which showed a high goodness-of-fit (R^2^ = 0.95) for the release exponent *n* = 0.561, implying anomalous (non-Fickian) drug release behavior due to drug diffusion and polymer matrix relaxation. It is worth mentioning that the NIR irradiation of CTX-CS-pDA@Ca^2 +^/GFT caused an increased K value (K = 0.312) (Table 2).

**Table 2:** Drug release kinetics and R^2^ values of CS-pDA@Ca^2+^/GFT, CTX-CS-pDA@Ca^2+^/GFT and CTX-CS-pDA@Ca^2+^/GFT + NIR

Kinetics model	CS-pDA@Ca^2+^/GFT		CTX-CS-pDA@Ca^2+^/GFT		CTX-CS-pDA@Ca^2+^/GFT + NIR	
	**R**^**2**^	K (constant)	**R**^**2**^	K (constant)	**R**^**2**^	K (constant)
Zero order model	0.89	6.42	0.83	0.511	0.78	4.62
First order model	0.91	0.63	0.87	0.511	0.82	0.47
Hixson Crowell	0.72	0.074	0.68	0.069	0.6	0.063
Higuchi model	0.97	0.312	0.94	0.284	0.90	0.256
Korsmeyer Peppas model	0.95	0.5 61	0.92	0.499	0.89	0.441

### In vitro studies

#### Cytotoxic effect of fabricated hydrogel

The cytotoxic property of GFT, CS-pDA@Ca^2 +^/GFT, CTX-CS-pDA@Ca^2 +^/GFT, and CTX-CS-pDA@Ca^2 +^/GFT + NIR hydrogel on KYSE-140 cells was evaluated using the CCK-8 assay. The results displayed that the CS-pDA hydrogel alone showed negligible cytotoxicity, confirming its good biocompatibility (Fig. S1. A). In contrast, free GFT exhibited a moderate inhibitory effect with an IC₅₀ value of approximately 60 µg/mL, while CS-pDA@Ca^2 +^/GFT displayed enhanced cytotoxicity with a lower IC₅₀ value of 50 µg/mL, demonstrating enhanced intracellular uptake and sustained release (Fig. S1. B & C). Further, the IC_50_ value of CTX-CS-pDA@CA^2+^/GFT was exhibited as 30 μg/mL, revealing that CTX functionalization promoted EGFR-mediated endocytosis and efficient drug accumulation in the cancer cells (Fig. S1.D). Furthermore, at the tested concentration of 50 μg/mL, the CTX-CS-pDA@Ca^2 +^/GFT hydrogel combined with NIR irradiation exhibited potent cytotoxicity, consistent with its low IC₅₀ value of 20 μg/mL, indicating a synergistic effect of photothermal therapy and chemotherapy. It can be observed from the findings that the cytotoxicity exhibited is because of the efficacy of the photothermal effect generated through pDA when exposed to NIR, hence causing local heating up, which enhances the permeability and diffusion of GFT [36]. Furthermore, the synergistic effect of the EGFR-targeted delivery system, apoptosis induced by calcium ions, and photothermal effect induced by NIR effectively inhibits KYSE-140 cell proliferation [37].

Viability and proliferation levels of KYSE-140 cells after treatment with NIR only, Ca^2+^, GFT, GFT + Ca^2+^, CS-pDA, CS-pDA@Ca^2 +^/GFT, CTX-CS-pDA@Ca^2 +^/GFT, and CTX-CS-pDA@Ca^2 +^/GFT+NIR hydrogel were determined through the EDU Apollo 488 assay. The presence of intense green fluorescence signals from both the control group and cells exposed to CS-pDA indicates that cell proliferation is occurring at an impressive rate, thus confirming that the delivery system is not cytotoxic to cancerous cells. Treatment with GFT resulted in some inhibition of cell proliferation, while CS-pDA@Ca^2 +^/GFT caused some inhibition of EDU-positive cells. Finally, CTX-modified hydrogel (CTX-CS-pDA@Ca^2 +^/GFT) inhibited cell viability and proliferation significantly, which can be attributed to increased EGFR-targeted uptake and GFT-mediated cytotoxicity (Fig S1 E & F).

Remarkably, CTXCSpDA@Ca^2 +^/GFT combined with NIR irradiation demonstrated the most significant inhibition of cell proliferation, with minimal EDU fluorescence intensity, indicating the synergistic contribution of photothermal and chemotherapeutic actions. These results corroborate previous studies where it was demonstrated that EGFR-targeted, near-infrared activated delivery systems could increase cytotoxic effects in cancer cells through receptor-mediated internalization and photothermal augmentation as well as polydopamine-coated chemophotothermal nanoparticles in esophageal cancer cells [38]. The research carried out by Chen et al. reported polydopamine-coated nanoparticles for synergistic chemo-photothermal therapy for EC, demonstrating effective tumor inhibition with photothermal energy [39]. NIR-activated EGFR-targeted nanosystems have been shown to facilitate rapid drug release and potent antitumor effects [40]. Additionally, calcium ions are key regulators of apoptosis through mitochondrial and endoplasmic reticulum pathways, providing mechanistic validation for the therapeutic strategy employed in this study [41].

#### Live/Dead and DAPI staining analysis of apoptosis in KYSE-140 cells treated with NIR-activated CTX-CS-pDA@Ca^2 +^/GFT hydrogel

**Fig. 5:**
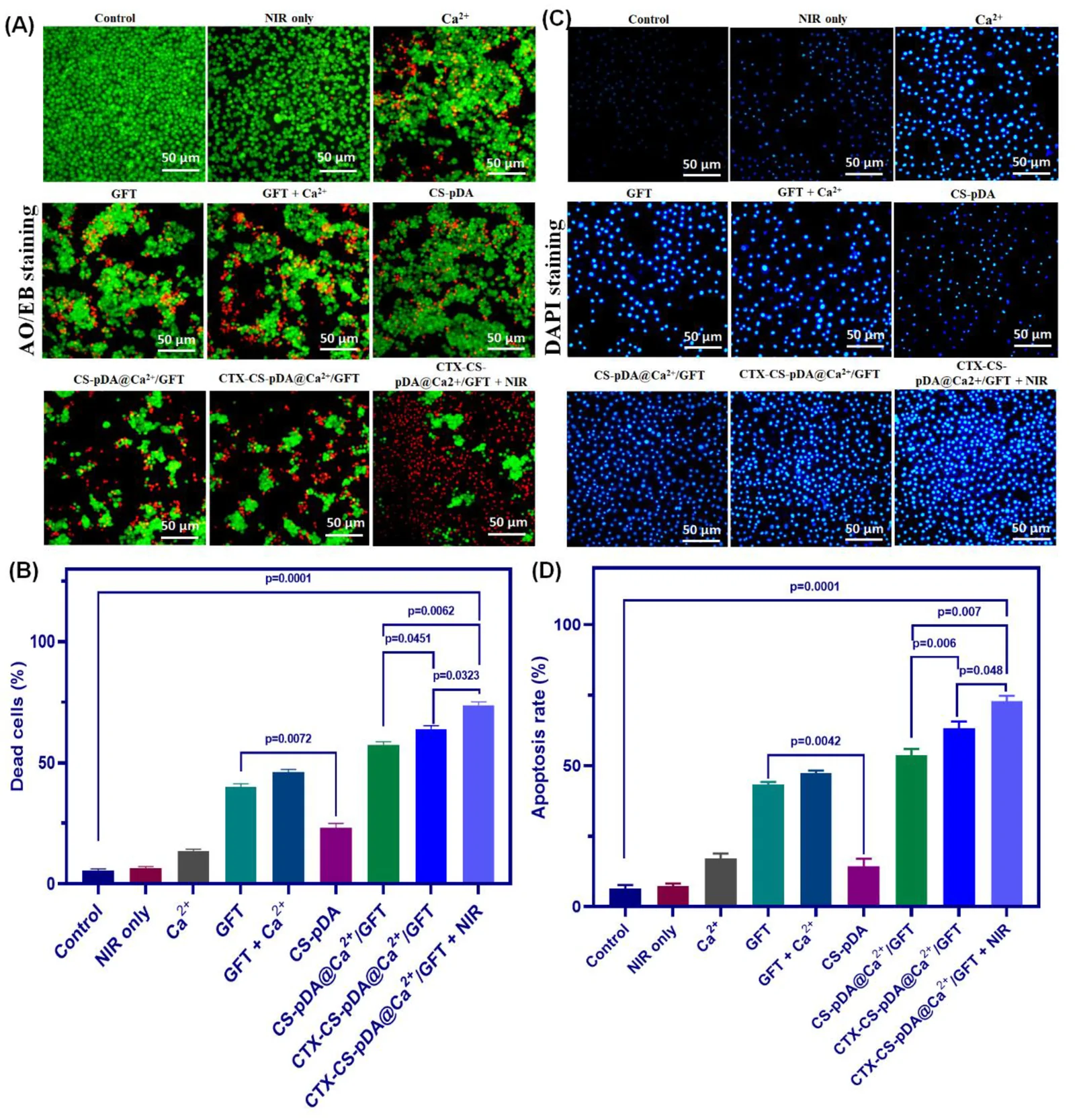
Evaluation of cytotoxicity and apoptosis in KYSE-140 esophageal cancer cells following treatment with different formulations. (**A**) Live/dead staining using AO/EB demonstrating cell viability (green: live cells; red: dead cells) across control, NIR only, Ca^2 +^, GFT, GFT + Ca^2 +^, CS-pDA, CS-pDA@Ca^2 +^/GFT, CTX-CS-pDA@Ca^2 +^/GFT, and CTX-CS-pDA@Ca^2 +^/GFT + NIR groups; (**B**) Quantitative analysis of dead cell percentage corresponding to AO/EB staining; (**C**) DAPI staining illustrating nuclear morphology changes, where treated groups show increased nuclear condensation and fragmentation compared to control; (**D**) Quantitative analysis of apoptosis rate (%) across different treatment groups. Data are presented as mean ± SD (*n* = 3). Statistical significance was determined by one-way ANOVA followed by Tukey’s post hoc test, and exact *p*-values are indicated in the figure

The cytotoxic effectiveness of the fabricated CTX-CS-pDA@Ca^2 +^/GFT hydrogel under NIR irradiation was evaluated using Live/Dead fluorescence staining (Fig. 5A). The quantitative study of the apoptosis ratio of KYSE-140 cells after treatment with NR only, Ca^2+^, GFT, GFT + Ca^2+^, CS-pDA, CS-pDA@Ca^2 +^/GFT, CTX-CS-pDA@Ca^2 +^/GFT and CTX-CS-pDA@Ca^2 +^/GFT + NIR is illustrated in Fig. 5B. In the untreated control group, the KYSE-140 cells displayed predominant green fluorescence, signifying intact cell membranes and increased cell viability.

For the untreated control group and for the NIR group, similar weak fluorescence was observed, which implies that NIR light itself is not cytotoxic [42]. Likewise, for the Ca^2 +^ group, the fluorescence is almost unchanged, proving that Ca^2 +^ by itself, at this particular concentration, does not have much effect on cell viability [43]. On the contrary, GFT, CTX-CS-pDA, and CTX-CS-pDA@Ca^2 +^/GFT induced an increase in red fluorescence with a corresponding decrease in green fluorescence, which shows that there was an increase in cell death. The combination of GFT + Ca^2 +^ demonstrated increased intensity of red fluorescence as opposed to GFT only, suggesting that Ca^2 +^ is able to enhance the cytotoxicity of GFT. Further, CTX-CS-pDA@Ca^2 +^/GFT with NIR showed a significant enhancement in red fluorescence, suggesting severe loss in cell viability (Fig. 5A & B). The elevated red fluorescence intensity observed in the NIR-exposed group indicates that near-infrared stimulation facilitated the effective release of GFT and Ca^2 +^ ions, leading to increased ROS generation and subsequent cellular membrane damage. Further evidence of apoptosis was provided by DAPI staining (Fig. 5C & D). The control, NIR-only, and Ca^2 +^ groups displayed uniformly stained, round nuclei, indicative of healthy morphology. In contrast, GFT, CTX-CS-pDA, and CTX-CS-pDA@Ca^2 +^/GFT-treated cells exhibited mild nuclear alterations, including chromatin condensation and slight fragmentation. The GFT + Ca^2 +^ group showed more noticeable nuclear deformation compared to GFT alone, suggesting enhanced apoptotic induction. However, cells treated with the NIR-triggered CTX-CS-pDA@Ca^2 +^/GFT hydrogel showed marked nuclear condensation, shrinkage, and fragmentation-hallmarks of apoptosis. These changes confirm the significant apoptotic potential of the NIR-responsive hydrogel system. The nuclear deformation and chromatin condensation observed upon NIR exposure suggest that GFT-induced EGFR inhibition, in combination with Ca^2 +^ -mediated mitochondrial stress and NIR-induced ROS production, collectively enhances apoptotic cell death [44]. These findings are in agreement with earlier studies reporting that NIR irradiation potentiates chemotherapeutic efficacy through photothermal and oxidative stress pathways, thereby promoting DNA damage and apoptosis in cancer cells [44, 45]. Thus, the Live/Dead and DAPI assays reveal that the NIR-activated CTX-CS-pDA@Ca^2 +^/GFT hydrogel significantly reduces cell viability and induces pronounced apoptotic features in KYSE-140 cells. These results suggest that the synergistic interplay of EGFR inhibition, calcium-induced stress, and NIR-triggered hyperthermia enhances the therapeutic potential of the engineered hydrogel for targeted esophageal cancer treatment.

#### Mitochondrial membrane potential analysis (MMP)

**Fig. 6:**
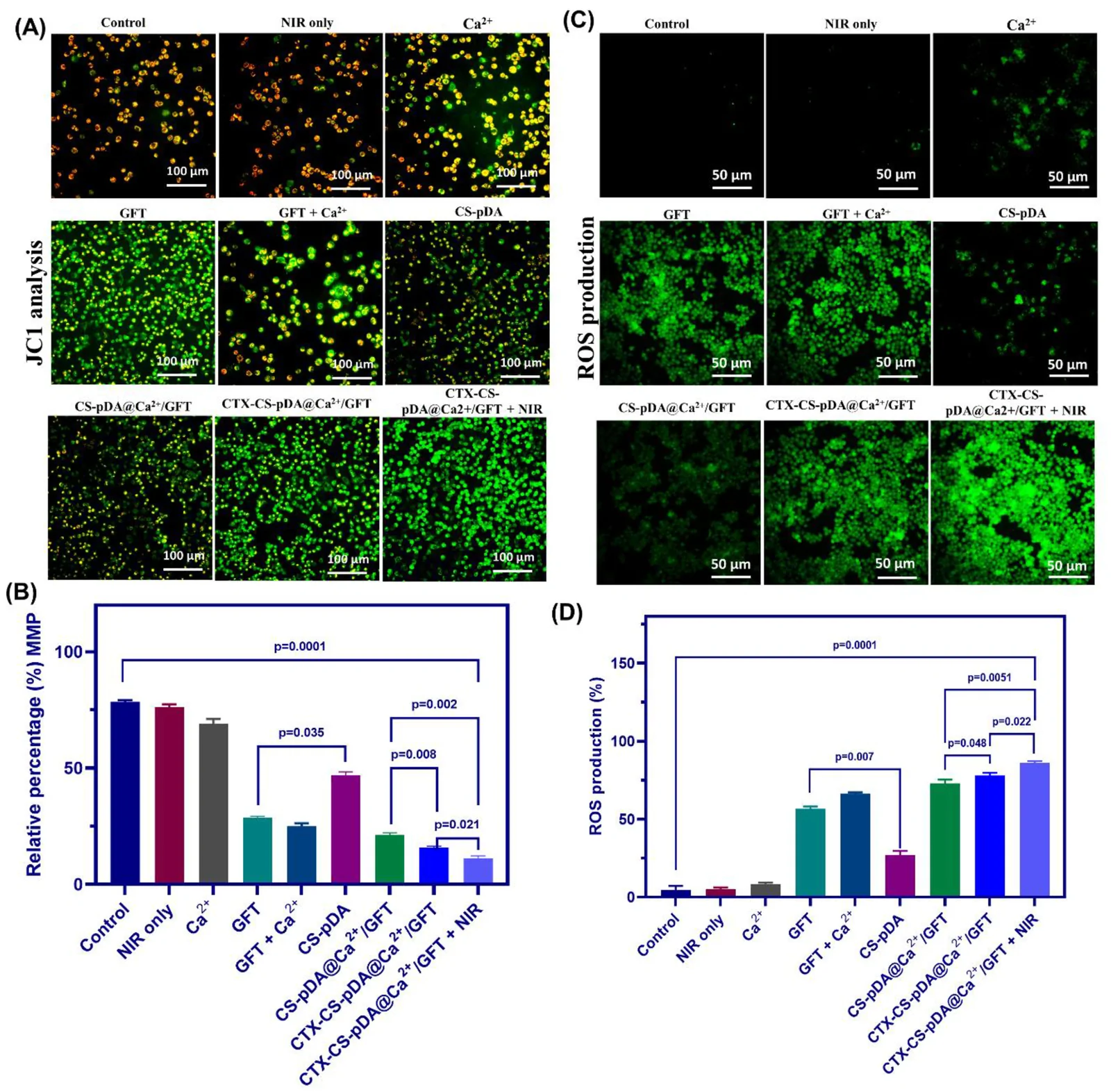
Assessment of mitochondrial membrane potential (MMP) and intracellular reactive oxygen species (ROS) generation in KYSE-140 cells following treatment with NIR alone, Ca^2+^, GFT, GFT + Ca^2+^, CS-pDA, CS-pDA@Ca^2 +^/GFT, CTX-CS-pDA@Ca^2 +^/GFT, and CTX-CS-pDA@Ca^2 +^/GFT + NIR. (**A**) JC-1 staining images illustrating changes in mitochondrial membrane potential across treatment groups; (**B**) Quantitative analysis of the red/green fluorescence ratio derived from JC-1 staining, indicating mitochondrial depolarization; (**C**) Representative fluorescence images of intracellular ROS generation detected using DCFH-DA staining after treatment; (**D**) Quantitative analysis of intracellular ROS levels based on DCFH-DA fluorescence intensity. Data are presented as mean ± SD (*n* = 3). Statistical significance was determined by one-way ANOVA followed by Tukey’s post hoc test, and exact *p*-values are indicated in the figure. Scale bars: 100 µm (**A**) and 50 µm (**C**)

A well-established indication of apoptosis in cancer cells is the collapse of the mitochondrial membrane potential (MMP), which is crucial for maintaining mitochondrial function and cellular homeostasis. In the present study, JC-1 staining was used to enumerate the changes in MMP after treatment with NIR-only, Ca^2 +^, GFT, GFT + Ca^2 +^, CS-pDA, CS-pDA@Ca^2 +^/GFT, and CTX-CS-pDA@Ca^2 +^/GFT, and CTX-CS-pDA@Ca^2 +^/GFT + NIR. As shown in Fig. 6A, control cells exhibit red-orange mixed fluorescence indicating intact and polarized mitochondria. Similar to that of control, NIR only exposed cells also exhibit similar fluorescence, suggesting that NIR irradiation alone exerts minimal effects on mitochondrial function unless combined with photothermal agents [46]. Ca^2+^ treated cells emit slight red fluorescence and minimal intensity in green fluorescence, indicating slight disruption of mitochondrial function.

Nevertheless, the cells treated with GFT exhibit a considerable decrease in red fluorescence and elevated intensity in green fluorescence, suggesting the initiation of mitochondrial membrane depolarization. Significantly, the GFT + Ca^2 +^ group exhibited a more pronounced red-to-green fluorescence shift compared to GFT alone, indicating that Ca^2 +^ enhances mitochondrial disruption and apoptotic signaling. Besides, cells exposed to the CTX-CS-pDA@Ca^2 +^/GFT exhibited a significant red-to-green fluorescence shift, indicating marked mitochondrial dysfunction. In terms of quantitative evaluation, it was found that NIR-mediated CTX-CS-pDA@Ca2+/GFT therapy was the most effective in terms of depolarizing the mitochondrial membrane (Fig. 6B). The damage to the mitochondria can be explained by the fact that the activation of NIR facilitates the release of GFT and Ca^2 +^ into the cell, resulting in oxidative stress and mitochondrial breakdown [47]. The increased depolarization could be attributed to ROS overproduction due to the effects of the GFT-EGFR blockade, mitochondrial overload because of Ca^2 +^, and enhanced NIR-induced photothermal therapy [48, 49]. This is consistent with other studies indicating that oxidative stress caused by NIR intensifies mitochondrial dysfunction, which promotes apoptosis in cancer cells [50]. In addition, NIR-induced Ca^2 +^ signaling has been demonstrated to disturb mitochondrial calcium regulation and consequently cause loss of mitochondrial membrane potential, triggering cell death by apoptosis [51]. Thus, the significant depolarization of cells treated with the combined effect of Ca^2 +^ and GFT under NIR irradiation provides clear evidence for the detrimental impact of the synergic effect of Ca^2 +^ and GFT under NIR stimulation on mitochondrial integrity, thus triggering apoptosis.

#### Intracellular measurement of ROS generation

ROS produced inside the cell was assessed by the staining of DCFH-DA in KYSE-140 cells after treatment with control, NIR only, Ca^2 +^, GFT, GFT + Ca^2 +^, CS-pDA, CS-pDA@Ca2+/GFT, CTX-CS-pDA@Ca2+/GFT and CTX-CS-pDA@Ca2+/GFT + NIR. The findings revealed distinct differences in ROS accumulation among the treatment groups (Fig. 6C). The KYSE-140 cells in control the group exhibit the very least fluorescence intensity, suggesting insignificant oxidative stress. Similarly, cells exposed to NIR alone exhibited fluorescence levels comparable to the control group, indicating negligible ROS generation in the absence of additional treatments. A slight increase in fluorescence intensity was observed in cells treated with Ca^2 +^ ions, indicating a relatively low level of oxidative stress. The elevated fluorescence intensity in the GFT + Ca^2 +^ group compared to GFT treatment alone reflects augmented oxidative stress mediated by Ca^2 +^. On the other hand, CTX-CS-pDA@Ca^2 +^/GFT exposed cells exhibited an appreciable increase in green fluorescence, which indicates that the synergistic effect of GFT and Ca^2 +^ release triggered significant oxidative stress. The NIR-treated CTX-CS-pDA@Ca^2 +^/GFT group shows higher levels of fluorescence intensity, indicating a considerable increase in ROS due to the photothermal effect. Quantitative analysis confirmed that ROS production was significantly higher in the NIR-irradiated group compared to the non-irradiated group (Fig. 6D). The increase in ROS generation is a result of the combined effects of CTX, GFT and Ca^2+^ presented in the hydrogel. The NIR light exposure increases the ability of the pDA matrix to absorb photothermal energy, thereby increasing ROS generation via hyperthermia [52]. Moreover, the released Ca^2 +^ ions interfere with mitochondrial function, thus leading to apoptosis via mitochondrial permeability transition [53, 54]. GFT contributes to this process by inhibiting EGFR-mediated pro-survival signaling, thereby sensitizing cancer cells to oxidative damage [55]. This oxidative stress causes impairment to the mitochondria and triggers apoptosis via the intrinsic apoptotic pathway, which is indicated by increased expression of cleaved caspases 3 and 9. This finding is consistent with recent studies proposing that ROS production and impaired mitochondrial function contribute to the effective apoptotic response triggered by the activation of NIR nanohybrids/hydrogels [53–55]. Consistent with this, Moon et al. (2023) & Gielecińska et al. (2024) demonstrated that Ca^2 +^ -induced oxidative stress is integral to apoptotic signaling, wherein ROS generation serves as a central mechanism underlying oxidation-induced cell death [41, 56].

#### Intracellular Ca^2 +^ accumulation induces apoptosis in KYSE-140 cells

**Fig. 7:**
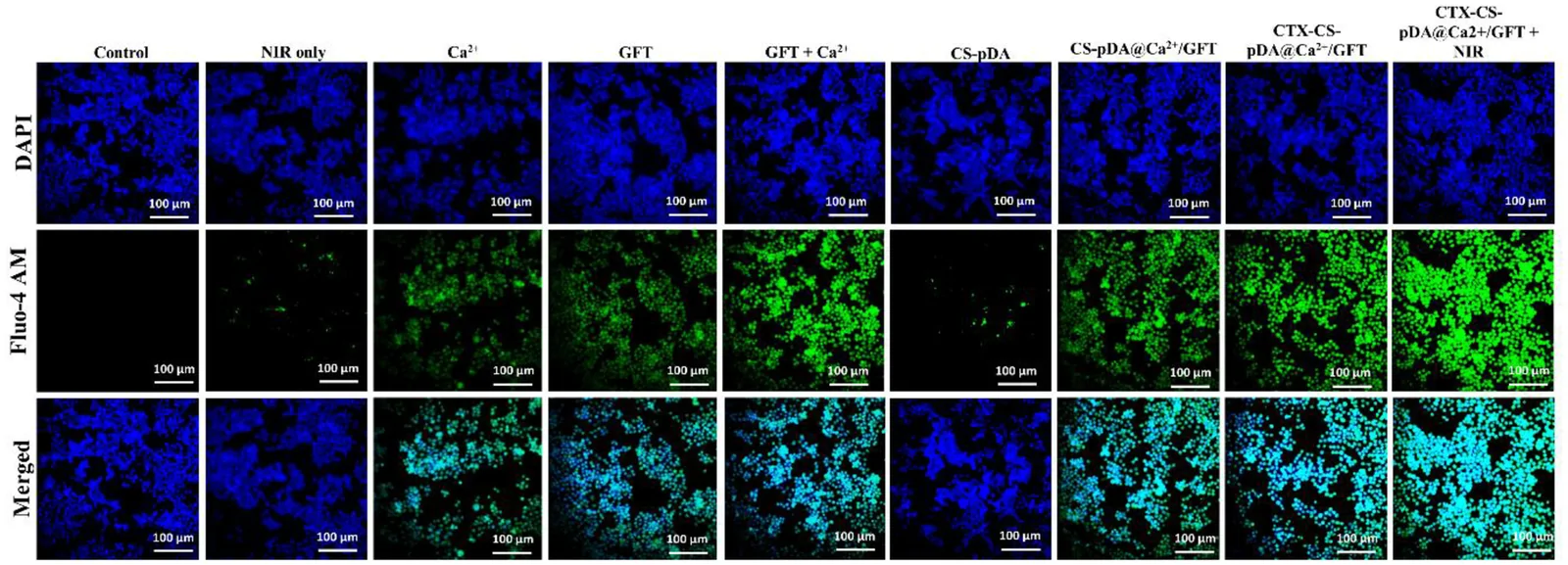
Representative fluorescence microscopy images showing intracellular Ca^2 +^ levels in KYSE-140 cells following different treatments. Cell nuclei were stained with DAPI (blue), and intracellular Ca^2 +^ was detected using the Fluo-4 AM probe (green). The merged images illustrate the co-localization of nuclei and Ca^2 +^ signals. Control (untreated), NIR-only, and CS-pDA groups exhibited minimal green fluorescence, indicating basal intracellular Ca^2 +^ levels. In contrast, Ca^2 +^, GFT, and GFT + Ca^2 +^ groups showed a moderate increase in fluorescence intensity. Notably, CS-pDA@Ca^2 +^/GFT and CTX-CS-pDA@Ca^2 +^/GFT groups demonstrated enhanced intracellular Ca^2 +^ accumulation. The highest fluorescence intensity was observed in the CTX-CS-pDA@Ca^2 +^/GFT + NIR group, indicating significant Ca^2 +^ overload induced by targeted delivery and NIR-triggered hydrogel activation. Scale bar: 100 μm

To investigate the calcium-dependant toxicity in KYSE-140 cells after treatment with fabricated hydrogel, intracellular Ca^2+^ levels were examined using Fluo-4 AM staining (Fig. 7). As shown in Fig. 7, the cells in the control and CS-pDA groups display least fluorescence intensity, suggesting basal intracellular Ca^2+^ production. Likely, the NIR-only group showed no significant alteration, confirming that irradiation alone does not induce Ca^2 +^ accumulation. In contrast, the cells subjected to Ca^2 +^ and GFT displayed a slight increase in fluorescence intensity, indicating an increase in intracellular Ca^2 +^ concentration due to the effect of stress caused by the calcium ions. In addition, the cells under the influence of GFT + Ca^2 +^ group exhibited an increase in fluorescence intensity, indicating the synergistic effect of GFT + Ca^2 +^ compounds on Ca^2 +^ accumulation.

In particular, intracellular Ca^2 +^ levels were significantly elevated in cells treated with CS-pDA@Ca^2 +^/GFT, proving that the fabricated hydrogel system successfully delivered and released Ca^2 +^. The CTX-CS-pDA@Ca^2 +^/GFT showed a considerable increase in fluorescence intensity due to possible receptor targeting and increased uptake. Amongst all tested, the CTX-CS-pDA@Ca^2 +^/GFT + NIR-exposed cells had maximum fluorescence intensity that implied the presence of a considerable amount of calcium in the cell. The increased Ca^2 +^ production under NIR irradiation may be related to quick hydrogel degradation and increased Ca^2+^ release. The presence of higher concentrations of intracellular Ca^2 +^ causes an imbalance in the homeostasis of the cells, thereby activating apoptosis due to mitochondrial dysfunction and the activation of downstream signaling pathways [57]. Furthermore, overloading the calcium ions within mitochondria is accomplished through mPTP opening, resulting in mitochondrial dysfunction along with the leakage of apoptosis-promoting elements such as cytochrome c, leading to caspase cascade activation and inducing cell death. A significant decrease in Bcl-2 levels, accompanied by increased expression of Bax and cleaved caspase-3, was observed in the CTX-CS-pDA@Ca^2 +^/GFT + NIR group. This suggests that the hydrogel induces apoptosis via synergistic chemotherapeutic effects and Ca^2 +^ -mediated stress. NIR activation enhances intracellular Ca^2 +^ accumulation, triggering mitochondrial dysfunction and ultimately leading to apoptosis [47].

The elevated intracellular Ca^2 +^ levels observed in this study suggest the involvement of Ca^2 +^ -mediated cell death pathways. It was noted in earlier studies that calcium overload disturbs intracellular ionic balance, causing an increase in calcium influx into mitochondria and the opening of mPTP with mitochondrial dysfunction [57, 58]. Such events facilitate the release of cytochrome c and caspase-dependent apoptosis. In addition, high concentrations of Ca^2 +^ have been shown to promote oxidative stress and further mitochondrial injury, promoting apoptosis [59]. Based on results obtained from this experiment, it is clearly shown that the significant increase of Ca^2 +^ inside cells, combined with apoptosis induction, plays an important role in mediating cell death. Although the direct involvement of specific molecular regulators of Ca^2 +^ - dependant cell death was not examined in this study, existing evidence supports the role of dysregulated intracellular Ca^2 +^ in driving Ca^2 +^ -mediated apoptotic cell death.

#### Cell migration and invasion assay

**Fig. 8:**
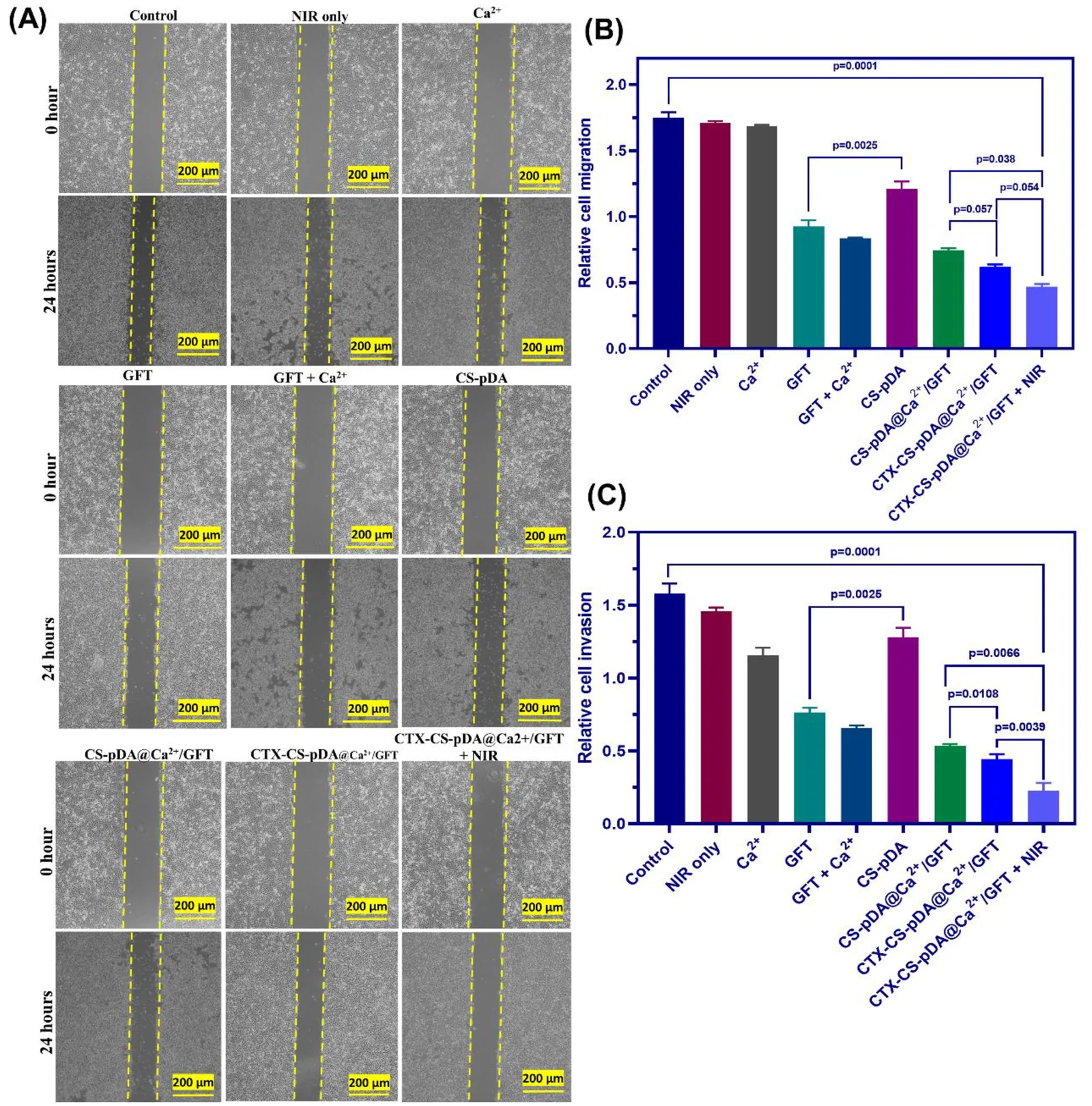
Evaluation of KYSE-140 cell migration and invasion following treatment with NIR only, Ca^2 +^, GFT, GFT + Ca^2 +^, CS–pDA, CS-pDA@Ca^2 +^/GFT, and CTX-CS-pDA@Ca^2 +^/GFT in the presence or absence of NIR irradiation. (**A**) Representative images of the wound healing (scratch) assay showing cell migration at 0 and 24 h after treatment; (**B**) Quantitative analysis of wound closure percentage, indicating the inhibitory effects of different treatments on KYSE-140 cell migration; (**D**) Quantitative analysis of cell invasion, demonstrating reduced invasive ability following treatment. Data are presented as mean ± SD (*n* = 3). Statistical significance was determined by one-way ANOVA followed by Tukey’s post hoc test, and exact *p*-values are indicated in the figure. Scale bar: 200 μm

As shown in Fig. 8A&B, the number of cells that migrated through the Matrigel-coated membrane was markedly reduced in the NIR-activated, dual-loaded hydrogel group compared with the untreated control. Cells treated with NIR alone exhibited migration patterns comparable to the control group, indicating that NIR irradiation does not significantly influence cell migration or invasion. Similarly, the Ca^2 +^ -treated group showed only a slight reduction in cell migration, suggesting minimal impact on invasive potential at the selected concentration.

In contrast, GFT-treated cells display a considerable decrease in cell migration, while further inhibition in cell migration was observed in cells treated with GFT + Ca^2+^. This was evidenced by the fact that, Ca^2+^ enhances the anti-migratory potential of GFT, indicating that Ca^2 +^ augments the anti-migratory effect of the drug. Among all treatment groups, the NIR-triggered CTX-CS-pDA@Ca^2 +^/GFT hydrogel demonstrated the most pronounced inhibition of invasion, confirming its strong anti-metastatic activity. Further, the Transwell invasion assay revealed that treatment with the NIR-irradiated CTX-CS-pDA@Ca^2 +^/GFT hydrogel substantially suppressed the invasive potential of KYSE-140 cells (Fig. 8C).

The observed inhibition in invasion may be attributed to the combined action of GFT and Ca^2+^ released from the hydrogel in the presence of NIR irradiation. Here, GFT, an EGFR tyrosine kinase inhibitor, is known to downregulate EGFR-mediated activation of PI3K/Akt and MAPK/ERK pathways, both of which play critical roles in tumor cell motility and invasion [60, 61]. Inhibition of EGFR signaling subsequently diminishes the expression of matrix metalloproteinases (MMP-2 and MMP-9), thereby triggering extracellular matrix degradation and cellular migration [62].

Correspondingly, Ca^2 +^ release from the hydrogel may enhance the inhibitory response by disrupting intracellular calcium homeostasis, leading to cytoskeletal reorganization and reduced motile capacity of cancer cells [63]. Additionally, NIR irradiation improves the localized release of both therapeutic components and enhances photothermal conversion by the pDA layer, which can further inhibit cytoskeletal activity and invasion-associated signaling [64]. Thus, it was evidenced that, NIR activated CTX-CS-pDA@Ca^2+^/GFT hydrogel significantly suppresses invasive and migratory behavior in EGFR-overexpressing EC cells via the synergistic inhibitory potential of EGFR signaling and calcium mediated-cytoskeletal modulation, revealing an effective treatment for preventing metastasis in ESCC.

#### Flow cytometric evaluation of apoptosis

**Fig. 9:**
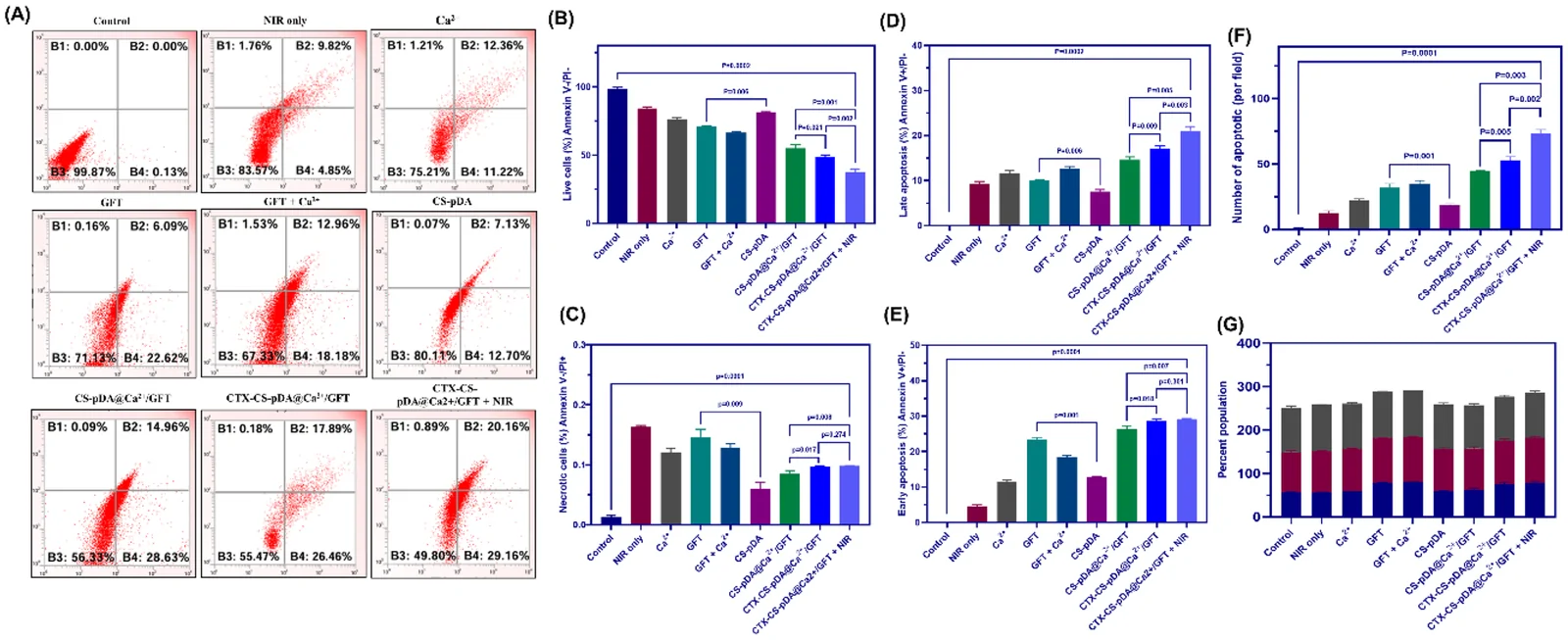
Flow cytometric analysis of apoptosis in KYSE-140 esophageal cancer cells following treatment with NIR only, Ca^2+^, GFT, GFT + Ca^2+^, CS-pDA, CS-pDA@Ca^2 +^/GFT, CTX-CS-pDA@Ca^2 +^/GFT, and CTX–CS–pDA@Ca^2 +^/GFT + NIR. (**A**) Representative Annexin V-FITC/PI flow cytometry dot plots showing the distribution of live, early apoptotic, late apoptotic, and necrotic cell populations among the different treatment groups; (**B-E**) Quantitative analysis of the percentages of live cells, early apoptotic cells, late apoptotic cells, and necrotic cells, respectively; (**F**) Quantification of apoptotic cells per field determined from fluorescence imaging; (**G**) Comparative distribution of total cell populations (live, early apoptotic and late apoptotic) for each treatment formulation. Data are presented as mean ± SD (*n* = 3). Statistical significance was determined by one-way ANOVA followed by Tukey’s post hoc test, and exact *p*-values are indicated in the figure

Flow cytometry was employed to quantify apoptotic events and analyze alterations in the cell cycle of KYSE-140 cells following treatment with control, NIR-only, Ca^2 +^, GFT, GFT + Ca^2 +^, CTX-CS-pDA, CTX-CS-pDA@Ca^2 +^/GFT, and CTX-CS-pDA@Ca^2 +^/GFT + NIR. Figure 9A depicts the viability and apoptosis levels in control (untreated) cells, which were viable with negligible signs of apoptosis, constituting the normal state of cells. In the case of the NIR-only group, there was almost an identical distribution of cells in relation to the control, hence demonstrating that laser light exposure did not contribute much to the process of apoptosis. Cells exposed to GFT showed a moderate increase in early apoptosis, suggesting that GFT has the ability to kill cells via inhibiting the EGFR pathway [65]. In addition, compared to cells treated with GFT alone, cells treated with GFT + Ca^2 +^ showed a greater number of apoptotic cells, indicating that Ca^2 +^ amplifies drug-induced apoptotic signals. Conversely, cells exposed to the CS-pDA group exhibited almost little apoptosis providing further evidence that the hydrogel matrix is biocompatible.

In contrast, the cells subjected to treatment with CTX-CS-pDA@Ca^2 +^/GFT exhibit a significant increase in both early and late apoptotic cell populations, revealing the synergistic cytotoxic effect of Ca^2+^-mediated stress and targeted GFT delivery (Fig. 9B–E). A significant decrease in viable cell percentage and an increase in total apoptosis was observed in the CTX-CS-pDA@Ca^2 +^/GFT + NIR group after NIR irradiation (Fig. 9F). This elevated apoptosis in cancer cells was noticed due to the action of NIR-induced hydrogel which allows targeted heating, enhanced drug release and increased ROS production [66, 67]. The increased ROS levels collapse the mitochondrial membrane potential and induce caspase activation, leading to apoptosis through both intrinsic and extrinsic pathways [68]. The results presented in Fig. 9G represent the overall cell distribution comprising the proportions of live cells, early apoptotic cells, and late apoptotic cells. The enhanced efficiency of the drug combination was demonstrated through the substantial reduction in the number of living cells and an increase in the late apoptotic cell population in the case of CTX-CS-pDA@Ca^2 +^/GFT + NIR. In this manner, the current study revealed the ability of CTX-CS-pDA@Ca^2 +^/GFT + NIR to promote apoptosis and cell cycle arrest through EGFR targeting using GFT.

### In vivo analysis

#### In vivo *evaluation of antitumor efficacy and systemic safety*

**Fig. 10:**
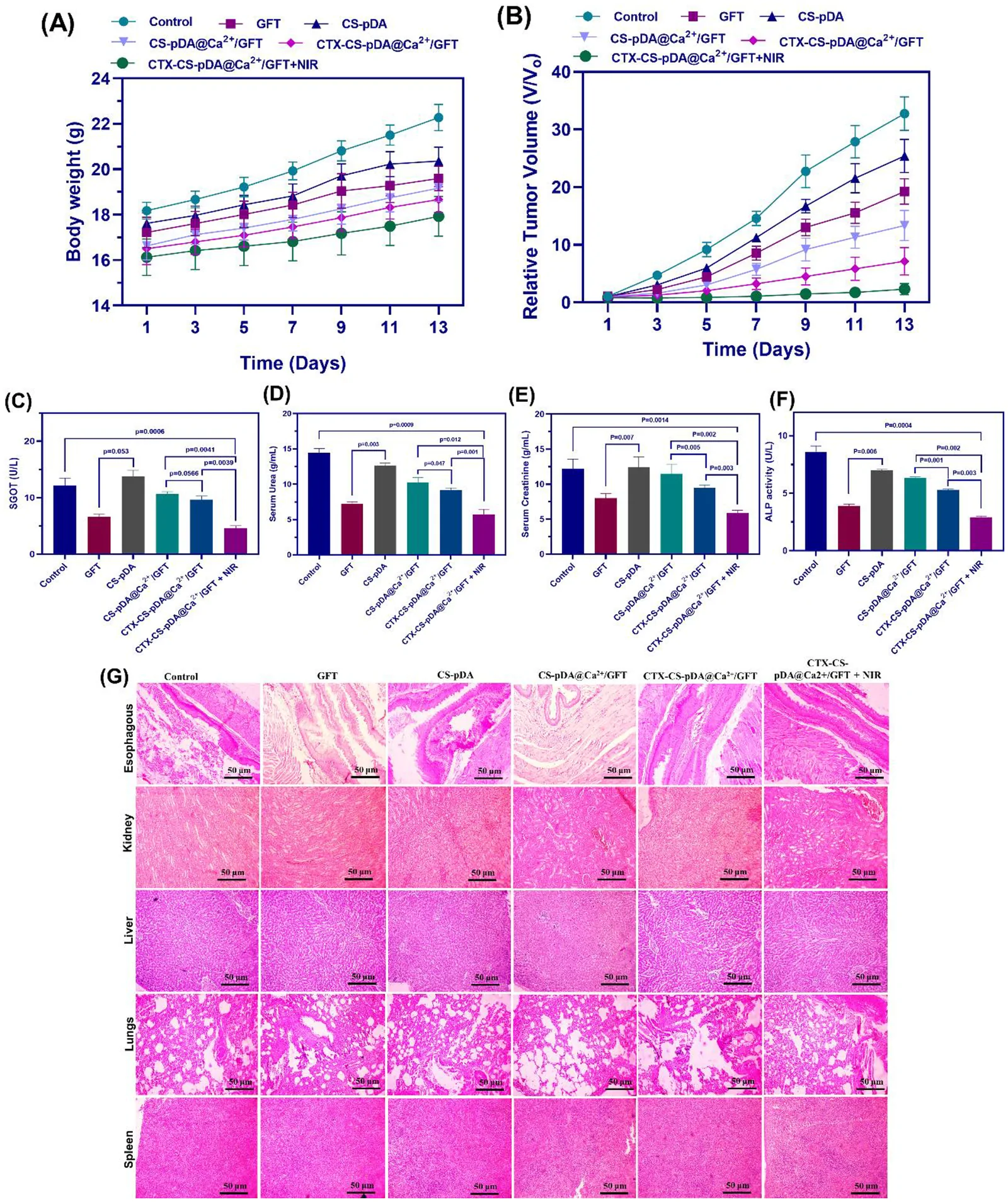
In vivo therapeutic efficacy and biosafety evaluation of different treatments in tumor-bearing mice. (**A**) Changes in body weight of mice over time, indicating overall systemic toxicity and treatment tolerability; (**B**) Relative tumor volume (V/V₀) measured at different time intervals, demonstrating significant tumor growth inhibition in treated groups. (**C-F**) Serum biochemical analysis including SGOT (**C**), serum urea (**D**), serum creatinine (**E**), and ALP activity (**F**), indicating hepatic and renal function across different treatment groups. (**G**) Histopathological examination (H&E staining) of major organs, including esophagous, kidney, liver, lungs, and spleen, showing tissue morphology and biosafety evaluation following treatment. Data are presented as mean ± SD (*n* = 6). Statistical significance was determined by one-way ANOVA followed by Tukey’s post hoc test, and exact *p*-values are indicated in the figure. Scale bar: 50 µm

The present investigation explored the in vivo antitumor potential and biosafety of the EGFR-targeted thermoresponsive CTX-CS-pDA@Ca^2 +^/GFT hydrogel in a tumor-bearing mouse model, both with and without NIR irradiation. During the experimental period, the body weight of the animals was continuously monitored to determine systemic toxicity of fabricated hydrogels. As depicted in Fig. 10A, there was no considerable reduction in body weight among all groups treated with hydrogel formulations, implying no substantial systemic adverse effect on their health status. Most importantly, the animals administered with CTX-CS-pDA@Ca^2 +^/GFT + NIR were seen with stable body weight, implying the successful implementation of this approach. Tumor size was recorded every other day through a digital caliper. As presented in Fig. 10B, there was an increased growth in tumor volume among animals in the control group over 13 days. On the contrary, the GFT, CTX-CS-pDA, and CTX-CS-pDA@Ca^2 +^/GFT treatment groups exhibited significant antitumor effects, indicating partial efficacy against cancer. In addition, the CTX-CS-pDA@Ca^2 +^/GFT + NIR group presented significantly decreased tumor size and weight in comparison to all other groups, highlighting the synergistic effect of NIR-activated drug release through the hydrogel system (Fig. 10B). The improved tumor inhibition achieved by CTX-CS-pDA@Ca^2 +^/GFT + NIR might be explained by the activation of the hydrogel triggered by NIR, which allowed for the controlled delivery of drugs, dysfunction in the mitochondria due to calcium ions, and increased production of ROS in the tumor environment, thereby enhancing EGFR inhibition by GFT.

Along with the effectiveness of the treatment, serum biochemical tests were carried out to study any possible liver toxicity and kidney toxicity. The tumor-bearing control group mice had elevated levels of SGOT, ALP, serum urea, and creatinine values, suggesting that the presence of tumors had caused liver and kidney damage (Fig. 10C–F). In contrast, the CTX-CS-pDA@Ca^2 +^/GFT + NIR group exhibited a marked reduction in these biochemical parameters, indicating improved safety and therapeutic efficacy. Collectively, these findings suggest that the CTX-CS-pDA@Ca^2 +^/GFT + NIR strategy represents a promising approach for the treatment of esophageal cancer, combining EGFR-targeted gefitinib delivery, Ca^2 +^ -mediated apoptosis, and NIR-induced hyperthermia to achieve enhanced antitumor activity with minimal toxicity. The observed biosafety profile of the fabricated hydrogel aligns well with earlier studies indicating that chitosan-derived hydrogels possess superior biocompatibility, biodegradability, and minimal toxicity, attributed to their natural composition and strong bioaffinity toward tissue systems [69]. In addition to that, pDA-incorporated hydrogels are known to display excellent cytocompatibility, adhesion capability, and antioxidative effects, leading to anti-inflammatory reactions and improved tissue affinity [70]. It is important to note that pDA is also employed for the same purpose as a photothermal agent, ensuring that it can sustain its temperature through exposure to NIR light and avoid any potential cytotoxic effects [71]. All these characteristics make the fabricated CTX-CS-pDA@Ca^2 +^/GFT hydrogel system an ideal candidate for targeted anticancer therapy.

#### Histopathology analysis for in vivo toxicity

In order to study the effectiveness and toxicology of the CTX-CS-pDA@Ca^2 +^/GFT hydrogel with and without NIR irradiation, the histopathological analysis was performed on the tissues of vital organs such as the liver, kidneys, spleen, lungs, and esophagus. The results showed that the CTX-CS-pDA@Ca^2 +^/GFT hydrogel, after activation by NIR, did not cause any significant changes in the normal organs. Esophagus, liver, kidney, spleen, and lung tissues from the NIR-triggered group maintained normal cellular architecture, with no significant inflammatory infiltration, necrosis, or vascular damage, suggesting that localized drug release and EGFR-targeted uptake successfully reduced systemic exposure (Fig. 10G). In contrast, the corresponding tumor tissues showed pronounced therapeutic damage, particularly in the CTX-CS-pDA@Ca^2 +^/GFT + NIR group. Morphological indicators of cytotoxicity-such as nuclear condensation, pyknosis, membrane disruption, and extensive apoptotic/necrotic zones, were markedly elevated compared with other treatment groups. These findings reveal that NIR activation enhances intratumoral drug release Ca^2 +^ enhances mitochondrial stress and EGFR-mediated uptake, collectively increasing tumor destruction. The enhanced therapeutic activity observed in the current study is reliable with previous reports indicating that NIR-triggered photothermal therapy significantly increases intratumoral drug release and triggers direct tumor cell damage through localized hyperthermia [72]. The deep tissue penetration capacity and high efficiency in producing thermal energy during NIR irradiation lead to denaturation of proteins, destruction of membranes, and initiation of immunogenic cell death [73]. In addition, it has been widely demonstrated that there is a clear function of calcium-induced mitochondrial stress in cancer treatments. Research shows that calcium ion inactivation caused by photothermal activation leads to mitochondria dysfunction, release of cytochromec, and induction of apoptosis via caspases [45].

Furthermore, CTX, an EGFR-targeting monoclonal antibody, has been reported to enhance therapeutic efficacy when combined with chemo-/phototherapy in EC. Recent clinical studies further suggest that CTX can improve treatment outcomes and prolong survival in patients with advanced esophageal carcinoma [74]. In addition, EGFR-targeted delivery systems have been widely reported to improve cellular uptake and therapeutic specificity in EGFR-overexpressing tumors. These EGFR-functionalized nanoparticles increase the occurrence of receptor-mediated endocytosis, thus improving the intracellular drug uptake and increasing anticancer activity [75]. In conjunction with NIR light treatment, this hydrogel shows marked elevation in apoptosis and inhibition of oncogenic pathway signaling [76]. Consequently, these histopathology results confirm that the NIR-sensitive CTX-CS-pDA@Ca2+/GFT hydrogel displays remarkable biocompatibility in normal cells yet exerts effective cytotoxicity against tumor cells, emphasizing their promising anti-cancer potential for EC.

#### Modulation of EGFR/AKT/PTEN signaling and apoptosis in tumor tissues

**Fig. 11:**
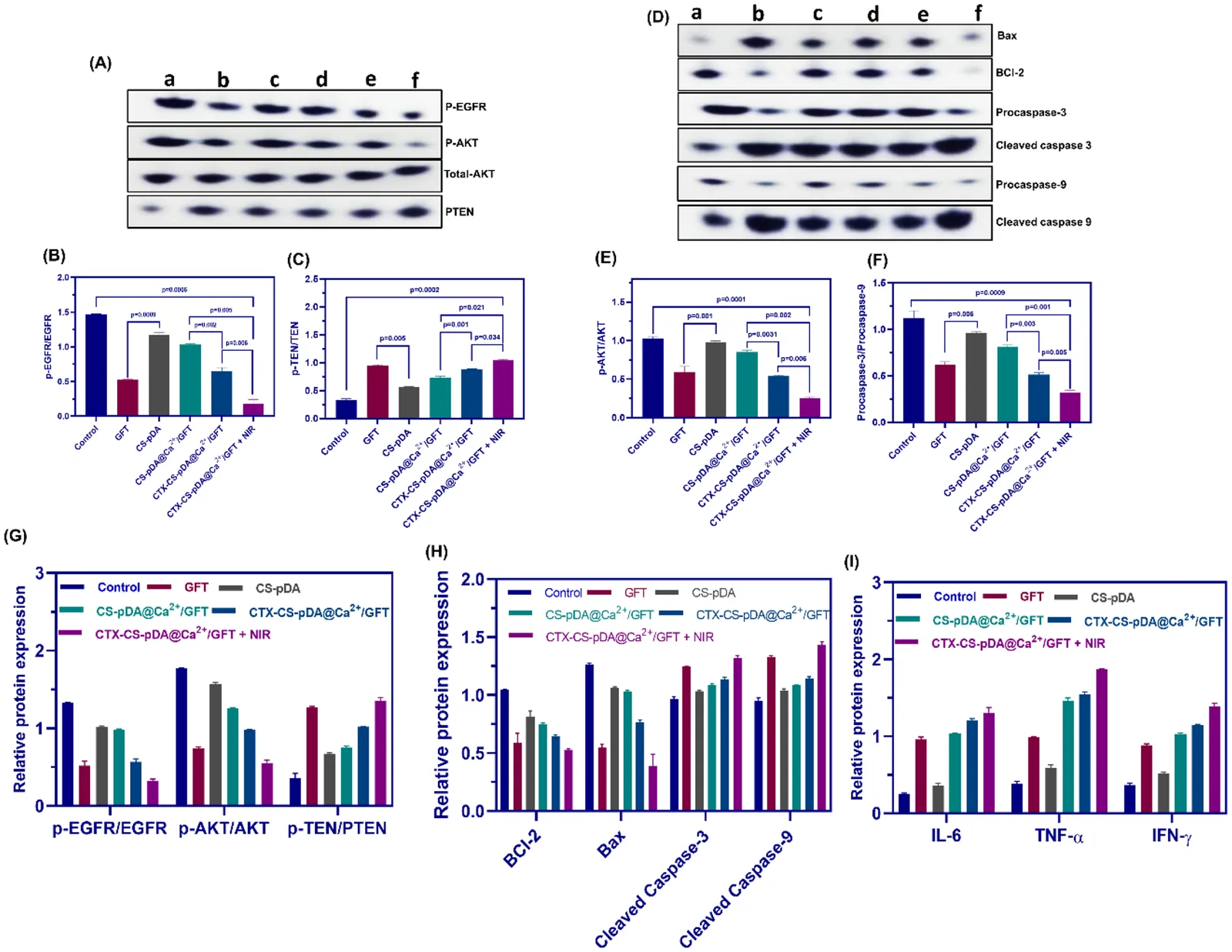
Evaluation of EGFR/AKT/PTEN signaling and apoptosis-related gene and protein expression in KYSE-140 esophageal carcinoma cells treated with GFT, CS-pDA, CS-pDA@Ca^2 +^/GFT, CTX-CS-pDA@Ca^2 +^/GFT, and CTX-CS-pDA@Ca^2 +^/GFT + NIR. (**A**) Representative Western blot images showing the expression of p-EGFR, p-AKT, total AKT, and PTEN across different treatment groups; (**B** & **C**) Quantitative analysis of p-EGFR/EGFR and PTEN expression levels; (**D**) Representative Western blot images of apoptosis-related proteins, including Bax, Bcl-2, procaspase-3, cleaved caspase-3, procaspase-9, and cleaved caspase-9; (**E** & **F**) Quantitative analysis of p-AKT/AKT and procaspase-3/cleaved caspase-3 expression ratios; (**G**) Relative protein expression levels of p-EGFR/EGFR, p-AKT/AKT, and p-TEN/PTEN; (**H**) Relative expression of apoptosis-related proteins (bcl-2, Bax, cleaved caspase-3, and cleaved caspase-9); (**I**) Relative expression of inflammatory cytokines (IL-6, TNF-α, and IFN-γ). Data are presented as mean ± SD (*n* = 6). Statistical significance was determined by one-way ANOVA followed by Tukey’s post hoc test, and exact *p*-values are indicated in the figure. (a- GFT, b-CS-pDA, c-CS-pDA@Ca^2 +^/GFT, d-CTX-CS-e-pDA@Ca^2 +^/GFT, and f-CTX-CS-pDA@Ca^2 +^/GFT + NIR)

Western blot and qRT-PCR analyses were conducted on tumor tissues isolated from the treated mice to determine how the NIR-responsive CTX-CS-pDA@Ca^2 +^/GFT hydrogel modulates oncogenic and apoptotic signalling pathways in vivo. Tumors obtained from animals receiving the dual-loaded hydrogel followed by NIR irradiation showed a pronounced decrease in phosphorylated EGFR (P-EGFR) and phosphorylated AKT (P-AKT) levels, while total EGFR and AKT expression remained largely unchanged (Fig. 11 A-C & G). The observed results reveal that the fabricated CTX-CS-pDA@Ca^2 +^/GFT + NIR effectively inhibits EGFR-mediated PI3K/AKT activation inside the tumor microenvironment, an important mechanism involved in the growth and development of EC cells [60, 77]. Moreover, a significant increase in the levels of PTEN protein was found in the tumors obtained from the NIR-activated hydrogel groups, indicating that PTEN activity was restored and providing negative feedback to the PI3K/AKT signaling pathway. Activation of PTEN also augments the antiproliferative and apoptotic effects of the EGFR inhibition approach [78]. Apoptosis-related protein profiling demonstrated a significant increase in the Bax/Bcl-2 ratio, accompanied by enhanced cleavage of caspase-3 and caspase-9, together with decreased levels of their inactive precursors (Fig. 11D-F & H). The corresponding uncropped Western blot images were provided as supplementary figure (Fig. S2). These results support the activation of the intrinsic mitochondrial apoptotic pathway in tumors [79]. The co-delivery of Ca^2 +^ and GFT, along with NIR hydrogel activation, may destabilize mitochondria, cause cytochrome c release, and facilitate caspase-dependent apoptosis. Collectively, molecular studies of extracted tumors have proved that the NIR activated CTX-CS-pDA@Ca^2 +^/GFT hydrogel could suppress EGFR/AKT oncogenic signaling while simultaneously activating PTEN inhibition and mitochondrial apoptosis. This dual targeting of pro-apoptotic and anti-survival pathways explains the effective antitumor properties achieved by this hydrogel platform for the treatment of EC.

#### Cytokine expression analysis by qRT-PCR

For examining the immunomodulatory activity of CTX-CTXPDA @Ca^2 +^/GFT + NIR, gene expressions of cytokines such as IL-6, TNF-α, and IFN-γ were examined in tumors by qRT-PCR analysis (Fig. 11I). It was observed that, in the control and CS-pDA group, all the above-mentioned cytokines were expressed at their base level, demonstrating that no immune response is present under healthy circumstances. In the CS-pDA treated group, there was no change in cytokine gene expression levels, showing the non-toxicity and non-immunostimulatory nature of CS-pDA. However, in case of GFT and Ca^2 +^ treatment, there was a significant increase in IL-6, TNF-α, and IFN-γ gene expressions due to therapeutic and ionic stresses on tumor cells, respectively. From Fig. 11I, it is evident that the synergy between GFT + Ca^2 +^ has shown great improvement in terms of the amount of cytokines produced by the body. Additionally, increased production of cytokines such as IL-6, TNF-α, and IFN-γ was noted in groups treated with CS-pDA@Ca2+/GFT hydrogel, in contrast to groups with single therapy. The levels of cytokine expression were even higher in the case of CTX-CS-pDA@Ca2+/GFT group, possibly because of EGFR-specific drug delivery, resulting in better internalization. Among all treated groups, the CTX-CS-pDA@Ca^2 +^/GFT + NIR group displayed significant upregulation in the expression level of those cytokines, indicating a pronounced immunomodulatory response. This is due to the NIR-triggered hydrogel activation, resulting in accelerated release of Ca^2 +^ and GFT, thereby intensifying tumor-associated stress signals. The corresponding uncropped Western blot images were provided as supplementary figure (Fig. S2).

From a mechanistic perspective, the increased production of cytokines such as IL-6 and TNF-α has been associated with inflammatory signaling pathways and tumor cell stress responses, whereas IFN-γ is an important cytokine involved in regulating immune responses and promoting apoptosis [80]. Based on the increase of cytokines, the hydrogel system developed not only displays cytotoxic action but also affects the tumor microenvironment via cytokine signaling pathways. However, it must be mentioned that the present analysis has only been focused on transcriptional level alterations, and further research at the protein level and immune cell migration tests are required to confirm immune activation. Collectively, all these results suggest that the CTX-conjugated Ca^2+^ loaded hydrogel has significant immunomodulation capabilities, which may help in boosting its efficacy against esophageal cancer.

## Conclusion

In this study, a novel NIR-responsive, cetuximab-functionalized chitosan-polydopamine hydrogel was developed for the co-delivery of Ca^2 +^ and gefitinib (GFT), targeting the epidermal growth factor receptor (EGFR) to achieve synergistic therapeutic effects against esophageal cancer. The hydrogel exhibited high drug loading capacity, stimulus-responsive release behavior, and excellent biocompatibility. In vitro studies demonstrated a significant enhancement in cytotoxicity, ROS generation, and apoptosis, which were further amplified under NIR irradiation. The combined action of GFT-mediated EGFR inhibition and Ca^2 +^ -mediated cellular stress markedly improved antitumor efficacy, while CTX functionalization facilitated targeted drug delivery. In addition, the system exhibited potential immunomodulatory effects associated with cytokine expression changes. Importantly, in vivo evaluation using a xenograft mouse model further validated the therapeutic potential of the developed hydrogel. The CTX-CS-pDA@Ca^2 +^/GFT + NIR treatment group exhibited significant tumor growth inhibition, accompanied by minimal changes in body weight and improved biochemical parameters, indicating low systemic toxicity. Histopathological analysis also confirmed the absence of major organ damage, supporting the biosafety of the formulation. These findings highlight the effectiveness of the hydrogel in achieving localized, sustained drug release with enhanced tumor suppression. Overall, the results suggest that this multifunctional hydrogel represents a promising platform for targeted and combinational cancer therapy. However, certain challenges remain to be addressed. The precise molecular mechanisms underlying Ca^2 +^ -mediated cell death and the observed cytokine-associated responses require further investigation. Additionally, comprehensive studies on long-term safety, pharmacokinetics, and clinical translation are necessary to fully establish its therapeutic potential.

## Electronic supplementary material

Below is the link to the electronic supplementary material.

Supplementary material 1

## Data Availability

The datasets used and/or analysed during the current study are available from the corresponding author on reasonable request.
