# Supplementary material for: NIR-responsive cetuximab-functionalized chitosan–polydopamine hydrogel Co- delivering Ca2+ and gefitinib for EGFR-targeted esophageal cancer therapy via Ca2+- mediated cytotoxicity and potential immunomodulatory effects
Source: J Biol Eng. 2026 Jul 22;20:151. doi: 10.1186/s13036-026-00726-w (PMC13679030; doi:10.1186/s13036-026-00726-w)
Supplement: Supplementary file 1 [file 13036_2026_726_MOESM1_ESM.docx]

**Figure. S1**

**Fig. S1.** Cytotoxicity and proliferative inhibition of GFT, CS–pDA, CS–pDA@Ca²⁺/GFT, and CTX–CS–pDA@Ca²⁺/GFT hydrogels in esophageal cancer cells.
(A–D) Dose-dependent cell viability of KYSE-140 cells treated with increasing concentrations (0–100 μg/mL) of (A) CS–pDA, (B) GFT, (C) CS–pDA@Ca²⁺/GFT, and (D) CTX–CS–pDA@Ca²⁺/GFT formulations, demonstrating enhanced cytotoxicity in the dual-loaded and CTX-functionalized systems; (E) Comparative cell proliferation (%) following treatment with NIR only, Ca^2+^, GFT, GFT + Ca^2+^, CS-pDA, CS-pDA@Ca²⁺/GFT, CTX-CS-pDA@Ca²⁺/GFT and CTX–CS–pDA@Ca²⁺/GFT + NIR; (F) Cell viability (%) of treated groups with NIR only, Ca^2+^, GFT, GFT + Ca^2+^, CS-pDA, CS-pDA@Ca²⁺/GFT, CTX-CS-pDA@Ca²⁺/GFT and CTX–CS–pDA@Ca²⁺/GFT + NIR. Data are presented as mean ± SD (n = 3). Statistical significance was determined by one-way ANOVA followed by Tukey’s post hoc test, and exact p-values are indicated in the figure.

**Figure. S2.**

**Fig. S2**. Uncropped Western blot images corresponding to the proteins analyzed in the study. Representative uncropped blots of (A) p-EGFR, p-AKT, total AKT, and PTEN; (B) Bax, Bcl-2, procaspase-3, cleaved caspase-3, procaspase-9, and cleaved caspase-9. Lane assignments for all blots are as follows: (a) Control, (b) GFT, (c) CS-pDA, (d) CTX-CS-pDA hydrogel, (e) CTX-CS-pDA@Ca²⁺/GFT, and (f) CTX-CS-pDA@Ca²⁺/GFT + NIR. These uncropped images correspond to the cropped Western blot data presented in Figure.11. of the main manuscript.
